# Metabolic and plasmid engineering to produce D-phenyllactic acid from glucose–xylose co-substrates in *Escherichia coli*

**DOI:** 10.1128/aem.02270-25

**Published:** 2025-12-03

**Authors:** Shuhei Noda, Yutaro Mori, Akihiko Kondo, Daisuke Nonaka, Mayumi Dainin, Ryosuke Fujiwara, Tsutomu Tanaka, Michihiro Araki, Tomokazu Shirai

**Affiliations:** 1 Graduate School of Science, Technology and Innovation, Kobe University 592891, Kobe, Japan; 2 PRESTO, Japan Science and Technology Agency (JST), Saitama, Japan; 3 Department of Chemical Science and Engineering, Graduate School of Engineering, Kobe University 542048, Kobe, Japan; 4 Center for Sustainable Resource Science, RIKEN, Yokohama, Kanagawa, Japan; 5 Graduate School of Pharmacy, Ritsumeikan University 197547, Kusatsu, Shiga, Japan; Danmarks Tekniske Universitet The Novo Nordisk Foundation Center for Biosustainability, Kgs. Lyngby, Denmark

**Keywords:** metabolic engineering, *Escherichia coli*, plasmid components, gene expression, xylose assimilation, D-phenyllactic acid

## Abstract

Plasmid DNA comprises various modules, including promoter and terminator regions, replication origins, and antibiotic resistance genes as selection markers. The 5′UTRs of the promoter and ribosome binding site (RBS) have been extensively studied, and multiple synthetic promoters and RBSs have been developed. In this study, we focused primarily on the components of plasmid vectors in *Escherichia coli*, excluding the 5′UTRs. Six replication origins and four selection markers were shuffled, resulting in the construction of 24 different plasmids. The gene encoding D-lactate dehydrogenase from *Cupriavidus necator* was cloned into each plasmid in this plasmid library, and the resulting 24 plasmids were evaluated based on D-phenyllactic acid production. The maximum concentration and yield of PheL from glucose were 5.45 g/L and 0.34 g/g, respectively. To the best of our knowledge, this yield represents the highest reported for PheL production. Furthermore, to examine the relationship between culture profiles and plasmid selection, clustering analysis was conducted, revealing the correlation between PheL production, and replication origins and selection markers. To demonstrate PheL production from biomass-derived resources, we also performed fermentation using mixed sugars of glucose and xylose with a metabolic pathway-compartmentalized *Escherichia coli* host. The gene cluster, including the Dahms pathway that converts xylose into acetyl-coenzyme A, was optimized, and the resulting transformant produced 3.5 g/L PheL with a glucose yield of 0.24 g/g.

**IMPORTANCE:**

As genetic and metabolic engineering has evolved, those technologies have enabled us to create synthetic microbes to produce chemicals of interest with high productivity. Although research on promoter regions and 5′ UTRs in plasmid DNA for the effective expression of heterologous genes has been widely conducted, there are few reports about the effect of replication origins and antibiotic resistance markers on production. In this study, we focused on the combination of those two factors and the effect on culture profiles. Using D-phenyllactic acid as the model chemical, the production and selection of replication origins and antibiotic resistance were evaluated. Additionally, we applied the metabolic pathway compartmentalized technology to the transformant showing the highest production, and D-phenyllactic acid production from the mixed sugars with glucose and xylose was demonstrated. This study provides new insights of the heterologous gene expression in microbial biosynthesis.

## INTRODUCTION

The development of metabolic engineering and synthetic biology has closely paralleled advancements in genetic engineering technologies. Innovations over the past few decades have enabled the construction of diverse artificial microbes. Numerous value-added chemical and pharmaceutical compounds can now be synthesized using model or versatile host strains, such as *Escherichia coli* and yeasts (1–4). Among these, *E. coli* is widely employed for product synthesis, and considerable research has focused on developing genetic engineering tools to manipulate *E. coli* and control the expression of heterologous genes. Promoter engineering and ribosome binding site (RBS) design, key drivers of gene expression, have become central topics in the genetic engineering of *E. coli* (5–8). Various promoter sequences have been utilized to produce different chemical products, and RBS libraries have been constructed in several studies. More recently, controllable gene expression systems using CRISPRa have been developed, establishing the engineering of 5′ untranslated regions (5′ UTRs) as a key strategy in bioproduction (9–11).

To express heterologous genes, two common strategies have been widely employed in the genetic engineering of *E. coli*: the use of plasmid DNA as a gene carrier and genome integration of genes of interest. Among these, plasmid-based expression is the most frequently adopted approach for introducing heterologous biosynthetic pathways. Plasmid DNA includes selection markers (e.g., antibiotic resistance genes) and replication origins in addition to the 5′UTRs previously mentioned. However, these components, excluding the promoter regions, have received limited attention in the metabolic engineering and synthetic biology of *E. coli*. Due to the lack of systematic strategies for optimizing gene expression within target biosynthetic pathways, researchers currently rely on the empirical testing of different gene orders to achieve maximal production yields (12–15). Our objective is to investigate the correlation between genetic information and target chemical production, thus providing clues for developing more systematic methodologies.

In microbial production research, novel systems have been developed to utilize multiple substrates efficiently and achieve high yields and titers of target chemicals. Li et al. (16) reported a coculture system using glucose and glycerol as substrates. In their study, multiple strains that produced essential factors for one another were co-cultivated in a single vessel, enabling self-regulated cell growth without requiring optimization of culture conditions. In particular, systems for the co-utilization of glucose and xylose are frequently reported. Zhang et al. (17) demonstrated the production of the value-added dicarboxylic acid, *cis*,*cis*-muconic acid, by employing a coculture of *E. coli* strains specialized for glucose or xylose assimilation, which resulted in an impressive yield. Conversely, a distinct strategy that uses multiple substrates for separate purposes within a single *E. coli* strain has also been reported (18). In that work, xylose supported cell growth, while glucose was channeled into the production of the target compound. The Dahms pathway was utilized for xylose assimilation, and two essential genes and two endogenous genes were overexpressed in *E. coli*. Thus, the use of multiple substrates has become one of the most actively studied strategies in microbial production.

Aromatic chemicals hold significant industrial and medical value. Several studies have explored microbial production of aromatic compounds using diverse host strains. Extension of the shikimate pathway is a widely adopted strategy to generate aromatic chemicals in microbes. Approaches to enhance this pathway have been well documented. The condensation reaction between phosphoenolpyruvate and erythrose 4-phosphate, along with the release of feedback inhibition at the enzymatic and transcriptional levels, is considered a critical point for pathway enhancement (19).

D-Phenyllactic acid (PheL) is an industrially significant aromatic chemical. It is applicable to the production of biodegradable polymers and exhibits antibacterial potential for use as a food additive due to its broad inhibitory effects on both Gram-negative and Gram-positive bacteria (20, 21). The microbial production of PheL has been reported in several studies, either through whole-cell biocatalysis converting L-phenylalanine or phenylpyruvic acid or via fermentation utilizing simple carbon sources as substrates (20–25).

In this study, four selection markers and six replication origins were shuffled in all possible combinations to investigate the effect of these plasmid vector components on D-PheL production. This approach aimed to explore how these often-overlooked vector components influence heterologous gene expression and metabolic output in engineered strains. Finally, optimization of the Dahms pathway gene cluster allowed us to produce D-PheL using mixed glucose and xylose substrates.

## RESULTS AND DISCUSSION

### Shuffling of selection markers and replication origins in the production of PheL

**Fig 1:**
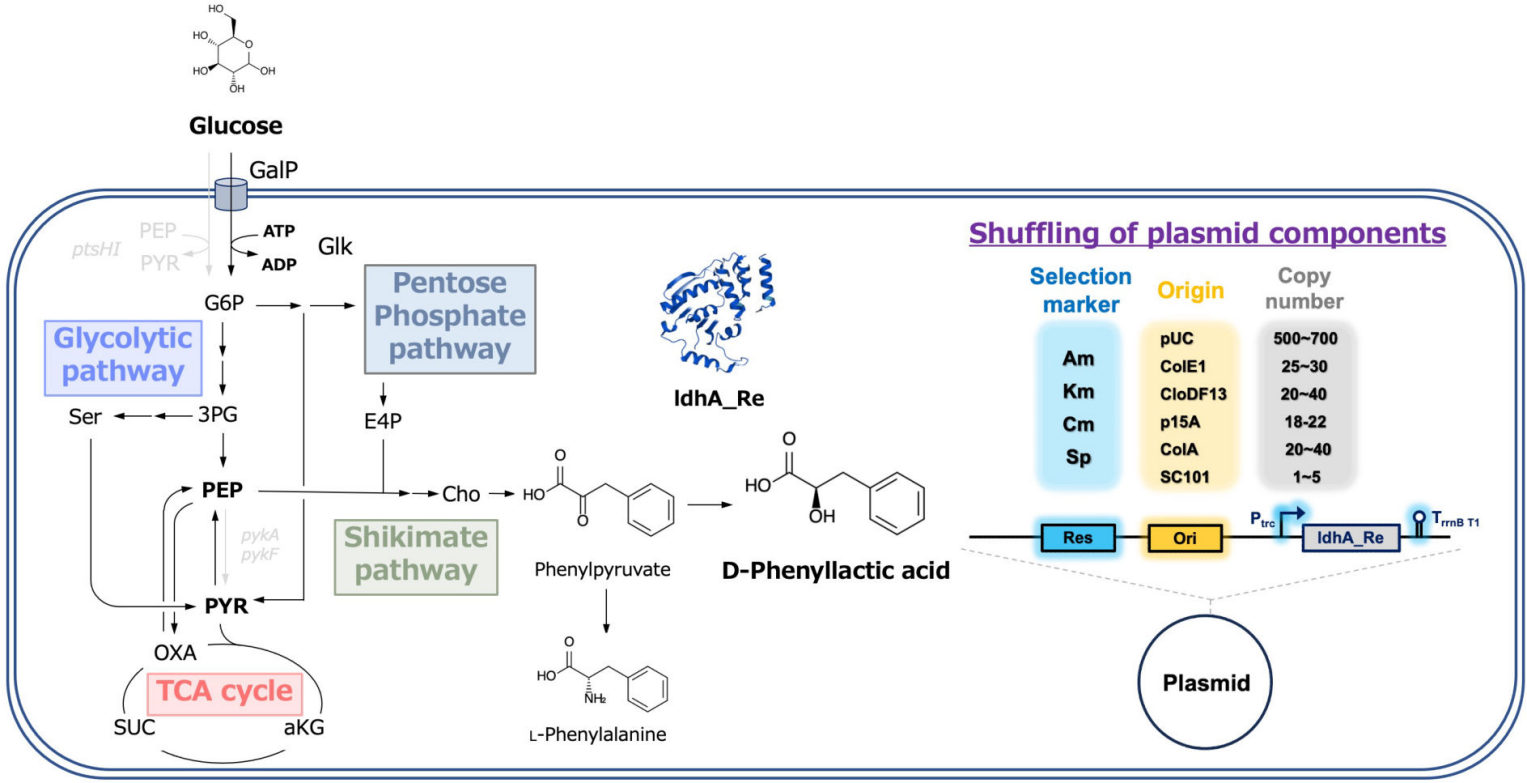
Metabolic pathway of D-phenyllactic acid from glucose and the concept of shuffling of plasmid components. Abbreviations: GalP, galactose permease; Glk, glucokinase; 3PG, 3-phosphoglyceric acid; Ser, L-serine; Cho, chorismate; E4P, erythrose-4-phosphate; G6P, glucose-6-phosphate; PYR, pyruvic acid; Oxa, oxaloacetate; SUC, succinic acid; aKG, alpha-ketoglutaric acid; PEP, phosphoenolpyruvate; ATP, adenosine triphosphate; ADP, adenosine diphosphate.

**Fig 2:**
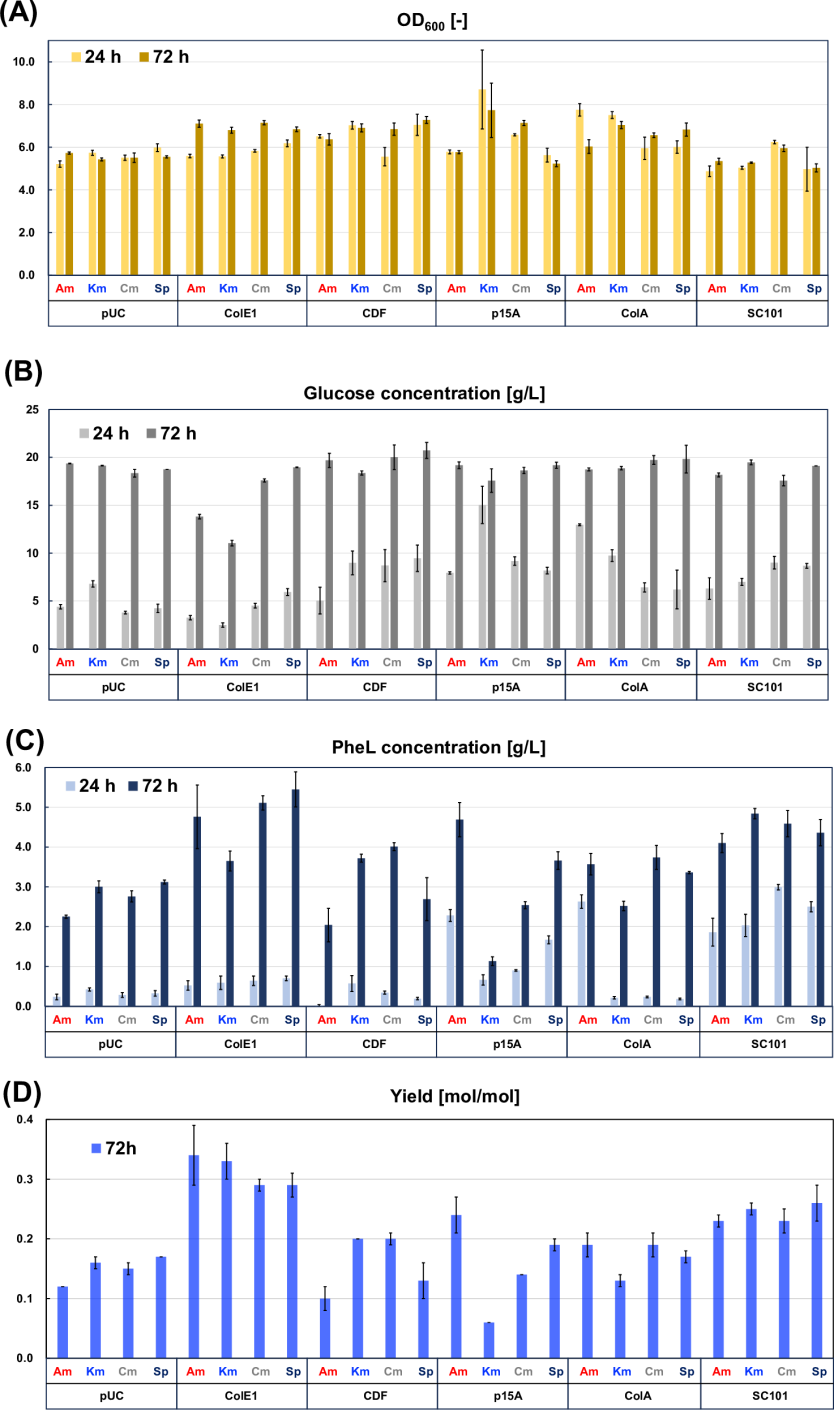
Culture profiles of 24 *E. coli* transformants harboring different plasmids for PheL production. (**A**) OD₆₀₀ values after 24 and 72 h of cultivation. (**B**) Glucose consumption after 24 and 72 h of cultivation. (**C**) PheL concentration after 24 and 72 h of cultivation. (**D**) Yield of PheL from glucose at 72 h. Data represent the mean ± standard deviation from three independent experiments.

We first focused on the components of gene expression vectors and investigated how these factors affected the production of the target compound. Using four selection markers and six replication origins, a plasmid library comprising 24 types of expression vectors was constructed (Fig. 1). The gene encoding D-lactate dehydrogenase from *Cupriavidus necator* (ldhA_Cu_) was subcloned into each plasmid under the control of the trc promoter. These vectors were introduced into CFT3, a strain that overproduces L-phenylalanine, resulting in 24 distinct gene expression patterns in PheL-producing *E. coli*. The *E. coli* strains were cultured in M9Y medium supplemented with 2% glucose, and their culture profiles were evaluated. Cell growth, glucose consumption, PheL production, and PheL yield are summarized in Fig. 2. Additional time-course details are provided in Fig. S1 and S2. These values varied significantly depending on the plasmid used. The maximum OD₆₀₀ values at 24 h of cultivation ranged from 4.7 for pSC101_Am to 8.7 for p15A_Km.

The final amount of consumed glucose was nearly the same across all strains, except for those using vectors carrying the ColE1 replication origin. Almost all strains in this experiment completely consumed the initially added glucose. However, the amount of glucose consumed within the first 24 h of cultivation varied depending on the vectors used in each culture.

Among the variables measured, PheL production and yield exhibited the greatest variation. For plasmids with the ColE1 origin, the maximum PheL concentration reached approximately 5.0 g/L, but the amount produced by 24 h remained below 1.0 g/L. Conversely, transformants carrying plasmids with the SC101 origin produced approximately 2.0 g/L of PheL by 24 h. In strains harboring plasmids derived from the p15A or ColA origins, PheL production levels differed depending on the antibiotic used as the selection marker.

**Fig 3:**
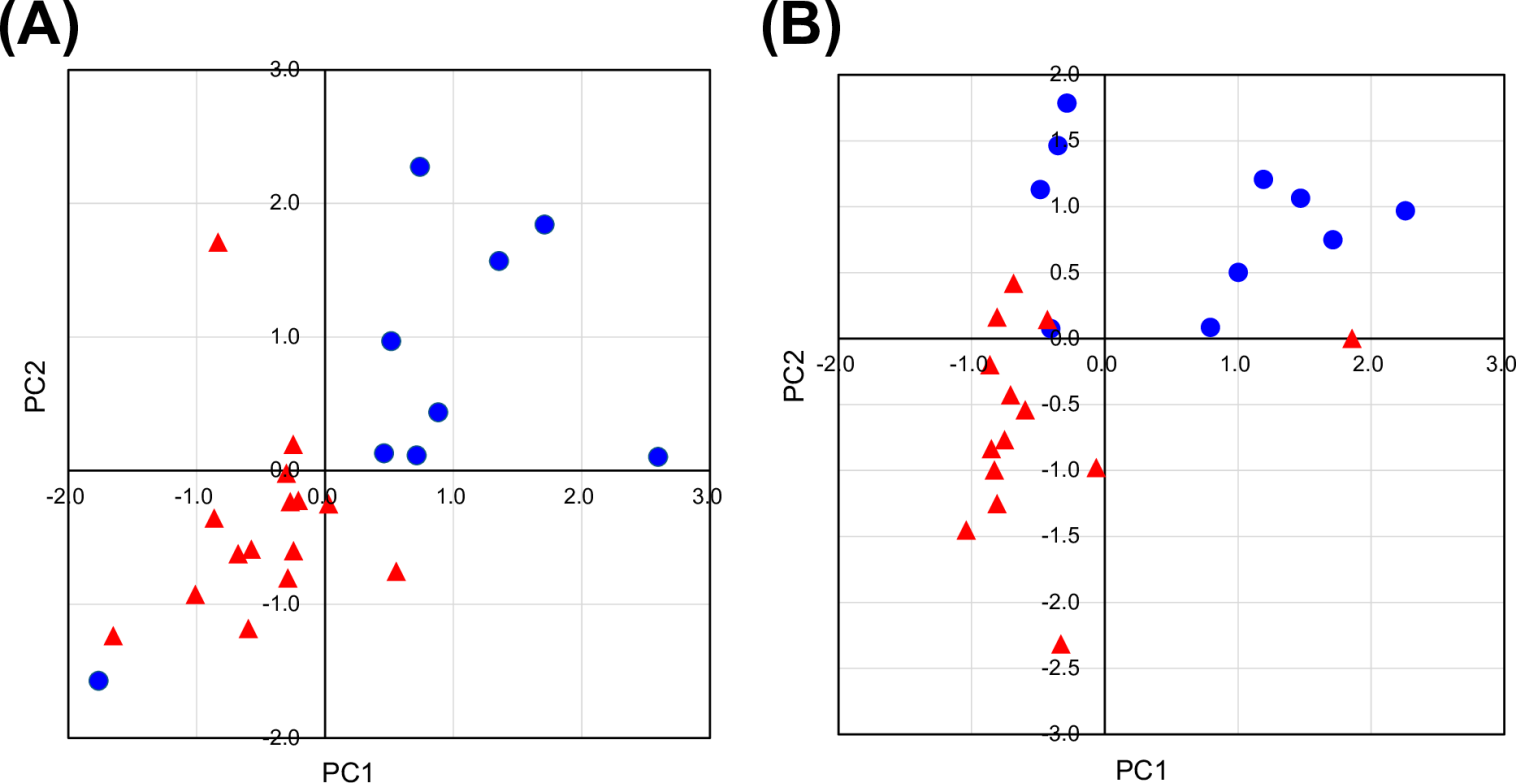
X-means clustering analysis of mRNA expression levels and culture profiles in 24 PheL-producing *E. coli* transformants. (**A**) Clustering results based on *ldhA_Cu_* mRNA expression levels after 24 and 48 h of cultivation in 24 transformants carrying different plasmid constructs. Blue symbols represent CFT3_pU_Cm, CFT3_pZE_Km, CFT3_pZE_Cm, CFT3_pCD_Cm, CFT3_pZA_Km, CFT3_pZA_Cm, CFT3_pSA_Km, and CFT3_pSA_Cm. Red symbols represent the remaining transformants. The plots data were summarized in Table S3. (**B**) Clustering results based on OD₆₀₀, glucose consumption, PheL concentration at 24 and 72 h, and PheL yield from glucose at 72 h. Blue symbols represent CFT3_pZE_Am, CFT3_pZE_Km, CFT3_pZE_Cm, CFT3_pZE_Sp, CFT3_pZA_Am, CFT3_pZA_Sp, CFT3_pSA_Am, CFT3_pSA_Km, CFT3_pSA_Cm, and CFT3_pSA_Sp. Red symbols represent the remaining transformants. The plots data were summarized in Table S4.

We investigated the mRNA expression levels of *ldhA_Cu_* after 24 and 48 h of cultivation (Fig. S4A)). Although the pUC origin is known for its super-high copy number, the expression levels of *ldhA_Cu_* mRNA were not higher than those observed with other plasmids. Similarly, no linear correlation was observed between mRNA expression levels and replication origin type across the transformants. To explore this further, clustering analysis using x-means was performed (Fig. 3A).

The 24 plasmids were grouped into two clusters based on their mRNA expression profiles at 24 and 48 h. With the exception of plasmids harboring the ColA origin, which showed relatively low *ldhA_Cu_* expression, one cluster with elevated mRNA levels was largely composed of constructs bearing Cm or Km resistance markers. However, plasmids containing Cm or Km resistance did not necessarily result in high PheL production. As shown in Fig. S4, the mRNA expression levels for the pUC and SC101 origins were not significantly different even though their copy numbers are known to be super-high and low, respectively. These results imply a correlation between mRNA expression levels and the amount of produced PheL although mRNA is not a complete predictor of the final titer. In addition to selecting the plasmids, post-transcriptional modification and regulation within the metabolic pathway are also important.

**Fig 4:**
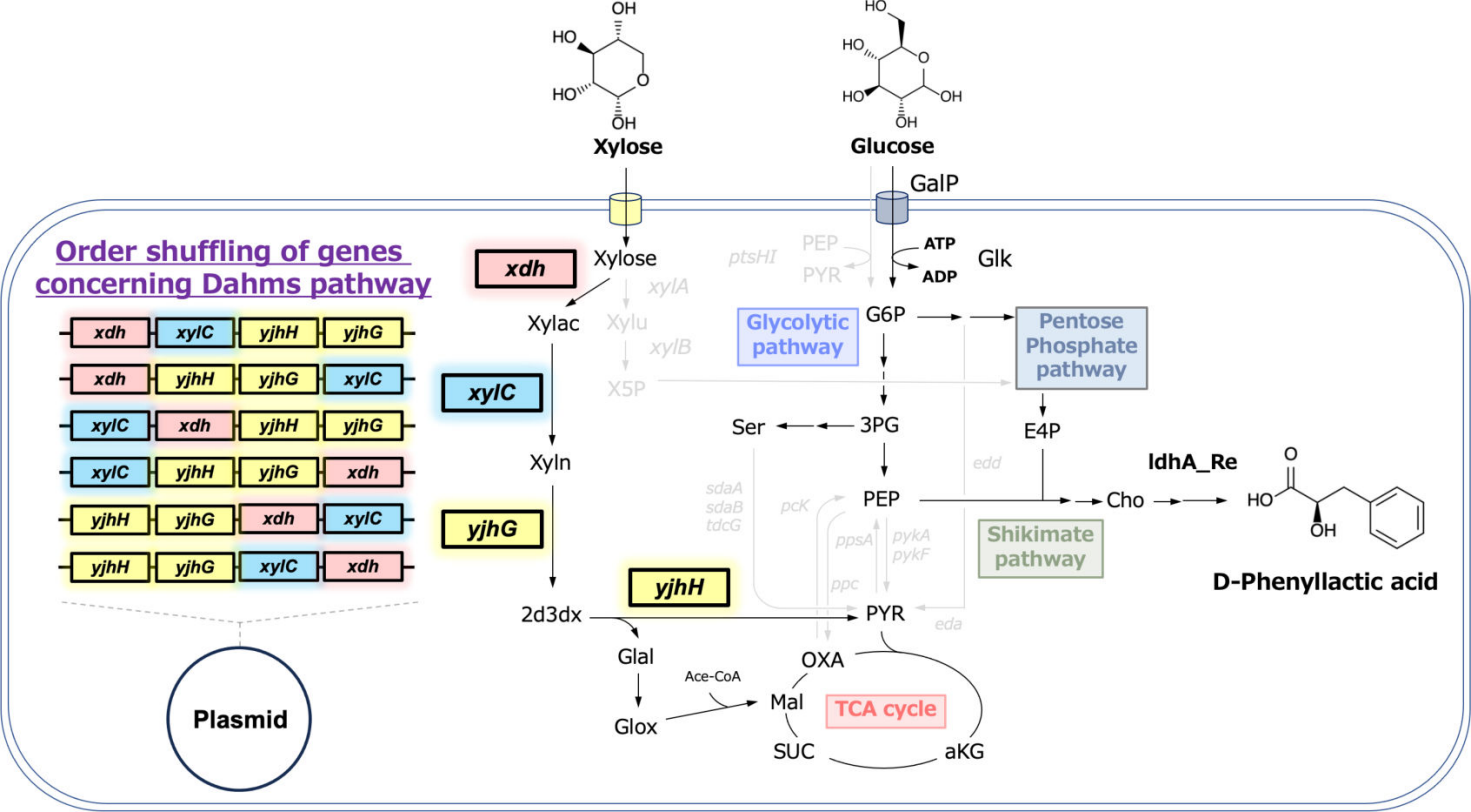
Metabolic pathway for D-phenyllactic acid production from glucose and xylose as co-substrates, and schematic overview of gene order shuffling within the Dahms pathway. Abbreviations: GalP, galactose permease; Glk, glucokinase; 3PG, 3-phosphoglycerate; Ser, L-serine; Cho, chorismate; E4P, erythrose4-phosphate; G6P, glucose6-phosphate; PYR, pyruvate; Oxa, oxaloacetate; SUC, succinate; aKG, α-ketoglutarate; PEP, phosphoenolpyruvate; ATP, adenosine triphosphate; ADP, adenosine diphosphate; Xylu, xylulose; X5P, xylulose 5-phosphate; Xylac, xylonolactone; Xyln, xylonate; 2d3dx, 2-keto-3-deoxy-xylonate; Glal, glycolaldehyde; Glox, glyoxylate; Ace-CoA, acetyl-CoA; Mal, malate.

We, therefore, investigated the amount of replicated plasmids in all 24 strains and calculated the concentration per unit of bacterial cell (Fig. S4B and C). According to the results in Fig. 3A and 4A, the mRNA expression in strains carrying plasmids with Cm or Km resistance markers was relatively higher than in other strains despite some exceptions. The concentration of each plasmid per unit of bacterial cell showed results similar to the mRNA expression levels, suggesting a certain correlation between the actual plasmid copy number and the mRNA expression level. Furthermore, the copy number of SC101 derivative plasmids, which are generally known as low-copy-number plasmids, was not extremely low compared to the other ones. These results might imply that the general information regarding the copy number of replication origins is unreliable.

Next, we performed clustering analysis focused solely on fermentation performance to examine the correlation between plasmid configuration and PheL production. As shown in Fig. 3B, this analysis, based on key culture metrics (OD₆₀₀, glucose consumption, PheL titer, and yield) but excluding *ldhA_Cu_* mRNA levels and replicated plasmid concentration, divided the 24 transformants into two groups: high and low PheL producers. Plasmids based on ColE1 and SC101 origins were associated with enhanced PheL production, regardless of the selection marker used.

We also performed clustering analysis that integrated *ldhA_Cu_* mRNA expression levels with the culture profile parameters, and the results are shown in Fig. S5. The 24 transformants were categorized into three clusters. However, no biologically meaningful patterns could be discerned from these groupings. It is possible that variation in the expression levels of heterologously expressed genes reduced the precision of the clustering analysis. In the case of using the ColA origin with Am or Cm resistance, the expression levels were relatively very low among the 24 strains created in this study; however, these strains produced 3.6 g/L and 3.7 g/L of PheL, respectively. This suggests that it is difficult to predict PheL productivity based on the level of mRNA expression alone, which corresponds to the results of the x-means clustering analysis incorporating mRNA expression levels in Fig. S5.

In previous studies, we employed these plasmid backbones for the production of other compounds. While SC101-based plasmids have not yielded high titers of alcohols, such as isobutanol or 2-phenylethanol (26, 27), they have proven suitable for the production of aromatic chemicals, such as salicylate and 4-nitrophenylalanine (28, 29). These findings suggest that no systematic correlation exists between plasmid features and target compound productivity. To develop a more rational framework for plasmid selection, future studies must consider additional factors, such as enzyme reaction mechanisms, protein expression burden, and gene sequence characteristics, alongside the plasmid components evaluated here. A more systematic approach requires the collection of large-scale data (big data) correlating plasmid choice with production yield, moving beyond the current trial-and-error dependency in metabolic engineering.

Despite the relatively low performance of batch cultures compared to fed-batch systems, the maximum yield achieved in this study was 0.34 g/g in the batch culture of CFT3_pZE_Am. This represents a 1.7-fold improvement over a previously reported yield (30), and the performance approaches that of other microbial platforms producing alcohols or organic acids under similar conditions (31–33). Recent PheL production metrics in batch cultures are summarized in Table S5.

### PheL production using metabolic pathway compartmentalized *E. coli* whose *xylAB* genes were inactivated

To produce PheL from mixed sugars of glucose and xylose, we utilized the metabolic pathway-compartmentalized *E. coli* strain CFT037 carrying pSAK-ldhA. pSAK-ldhA was previously classified in the high-performance plasmid cluster, and pZE12-x was employed to introduce genes encoding the Dahms pathway for xylose assimilation (18). Excluding the Dahms pathway, there are two major xylose assimilation routes: one uses xylose isomerase, and the other uses the combined pathway of xylose reductase and xylitol dehydrogenase. In these major pathways, xylose is incorporated into the pentose phosphate pathway, whereas the Dahms pathway generates pyruvate and glyoxylate as precursors for the TCA cycle. In the present study, we focused on the Dahms pathway to enhance the production and yield of PheL from glucose during cultivation using mixed glucose and xylose substrates.

**Fig 5:**
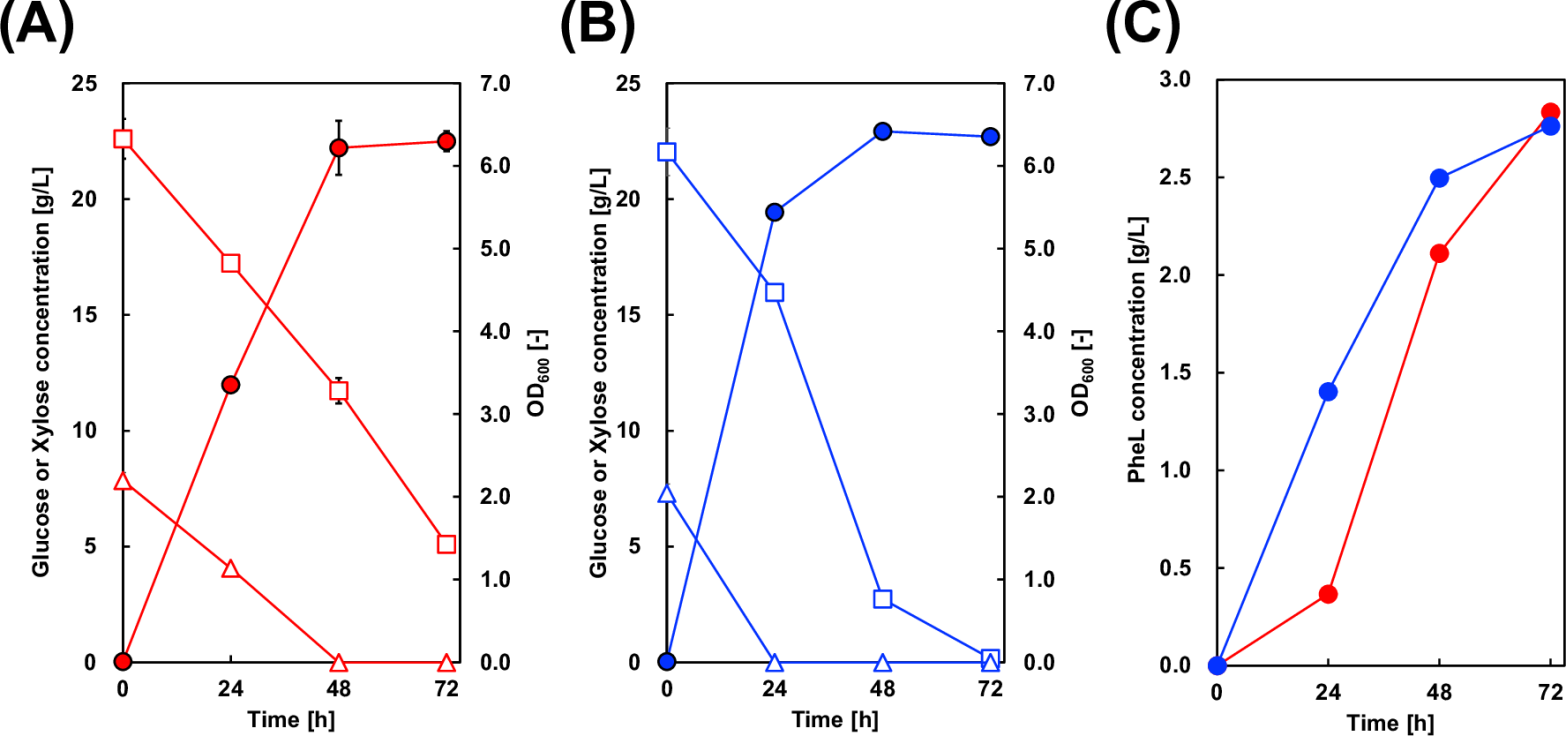
Culture profiles of C37_12_pS and C37_12x_pS. (**A, B**) Time-courses of cell growth (circles), glucose concentration (squares), and xylose concentration (triangles) for C37_12_pS without the Dahms pathway and C37_12x_pS with that, respectively. (**C**) Time-course of PheL production by both strains. Red and blue symbols represent C37_12_pS and C37_12x_pS, respectively. Data are shown as the mean ± standard deviation from three independent experiments.

The metabolic configuration of the C37_12x_pS strain is illustrated in Fig. 4. C37_12x_pS cells were cultured in M9Y medium containing both glucose and xylose, and the culture profiles were analyzed (Fig. 5). Figure 5A presents the sugar consumption and growth profile of the control strain lacking the Dahms pathway genes, while Fig. 5B shows the data for the C37_12x_pS strain. Although the C37_12x_pS strain exhibited faster uptake rates for both glucose and xylose than the control, the final levels of xylose assimilation and PheL production were similar between the two strains.

Two modifications were made to the metabolic pathway in the C37_12x_pS strain. First, the native *xylAB*-encoded xylose assimilation pathway was inactivated, thereby blocking carbon flux from xylose to the pentose phosphate pathway.

Second, the *sdaB* and *tdcG* genes, which encode serine deaminases, were also inactivated based on our previous observation that these genes were unexpectedly activated under certain conditions (29). In that previous report, *E. coli* strains with a compartmentalized metabolic pathway were used to produce salicylate and maleate. The biosynthesis pathways of both chemicals involve pyruvate formation in the final step, and the TCA cycle was driven by the resulting pyruvate. In the case of salicylate production using CFT575 (where *sdaB* and *tdcG* were not inactivated), the yield from glucose reached 50% (mol/mol), which was 80% of the theoretical maximum. However, the yield was only 13% for maleate production. These results suggest that the activation of *sdaB* and *tdcG* is triggered depending on the introduced gene or gene clusters.

**Fig 6:**
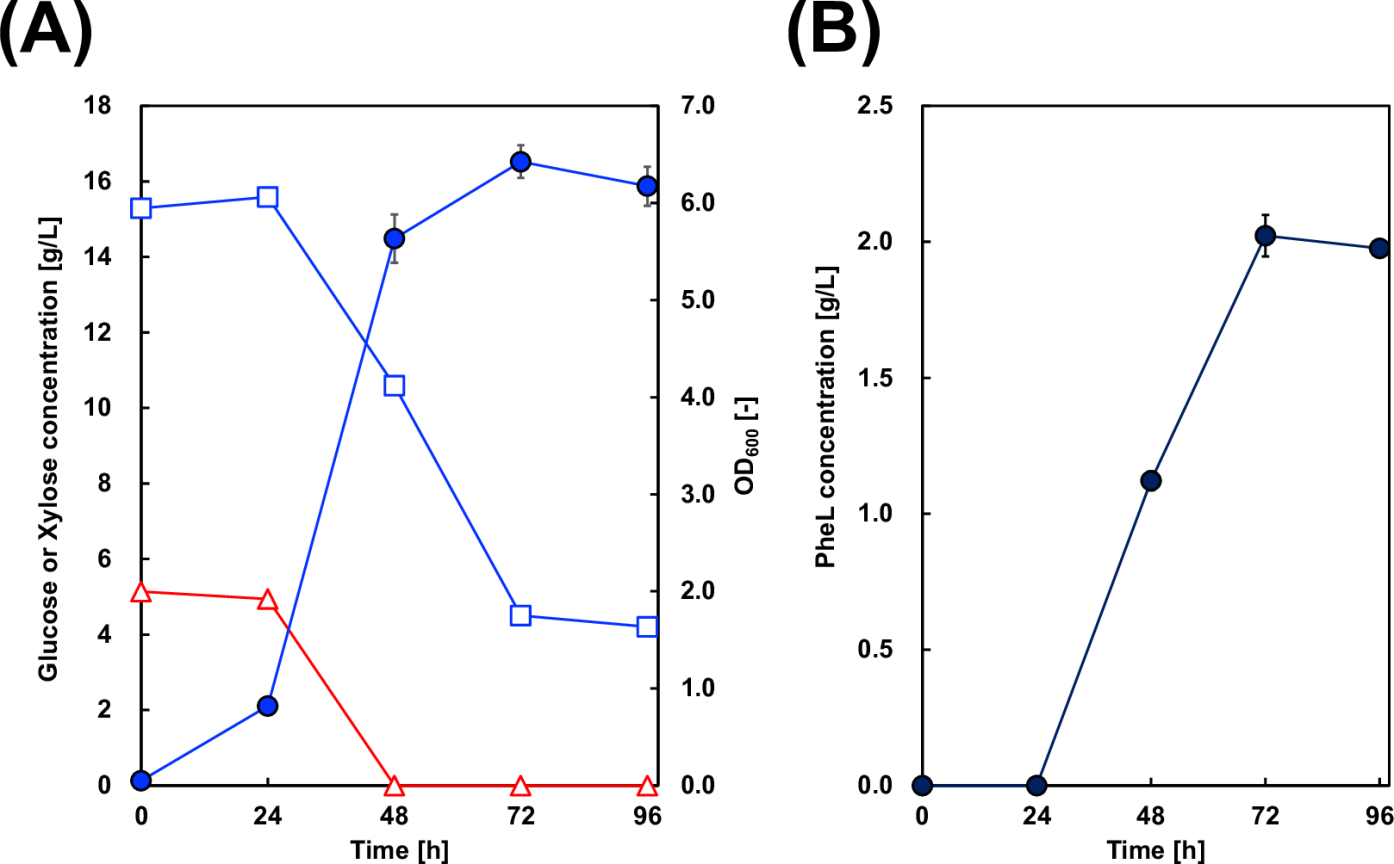
Culture profiles of C40_12x_pS. (**A**) Time-course profiles showing bacterial cell growth (circles), glucose concentration (squares), and xylose concentration (triangles). (**B**) Time-course of D-phenyllactic acid (PheL) production. All values are expressed as the mean ± standard deviation from three independent experiments.

To avoid this unexpected gene activation, these three genes and the *xylAB* gene cluster (*sdaB*, *tdcG*, and *xylAB*) were deleted to construct strain C40_12pS for improved PheL production from glucose and xylose. C40_12pS cells were cultured in M9Y medium containing mixed sugars, and the culture profiles were analyzed (Fig. 6). Although xylose was completely consumed, the uptake rate was lower than that observed in C37_12x_pS. This might be attributed to an imbalance in the expression of genes for PheL production and xylose assimilation. Furthermore, C40_12pS cells assimilated only 10 g/L of glucose, and PheL production reached just 2.0 g/L, which was lower than that of both the C37_12x_pS and control strains.

### Gene order shuffling of the Dahms pathway to enhance PheL production

As described above, strict metabolic compartmentalization, such as inactivating *xylAB* and genes encoding serine deaminases, did not enhance glucose utilization or PheL production. Therefore, we focused on optimizing gene expression balance within the Dahms pathway to improve metabolite flux toward PheL in engineered *E. coli*.

To achieve this, two single genes (*xdh* and *xylC*) and one gene cluster (*yjhHG*) from the Dahms pathway were shuffled into six tandem combinations (Fig. 4). The *yjhHG* operon, sourced from the *E. coli* genome, was treated as a functional gene cluster. Although we attempted to construct five distinct *E. coli* strains (excluding the previously evaluated C40_12pS), one transformant carrying the *yjhHG–xdh–xylC* arrangement in the pZE12MCS vector could not be obtained.

**Fig 7:**
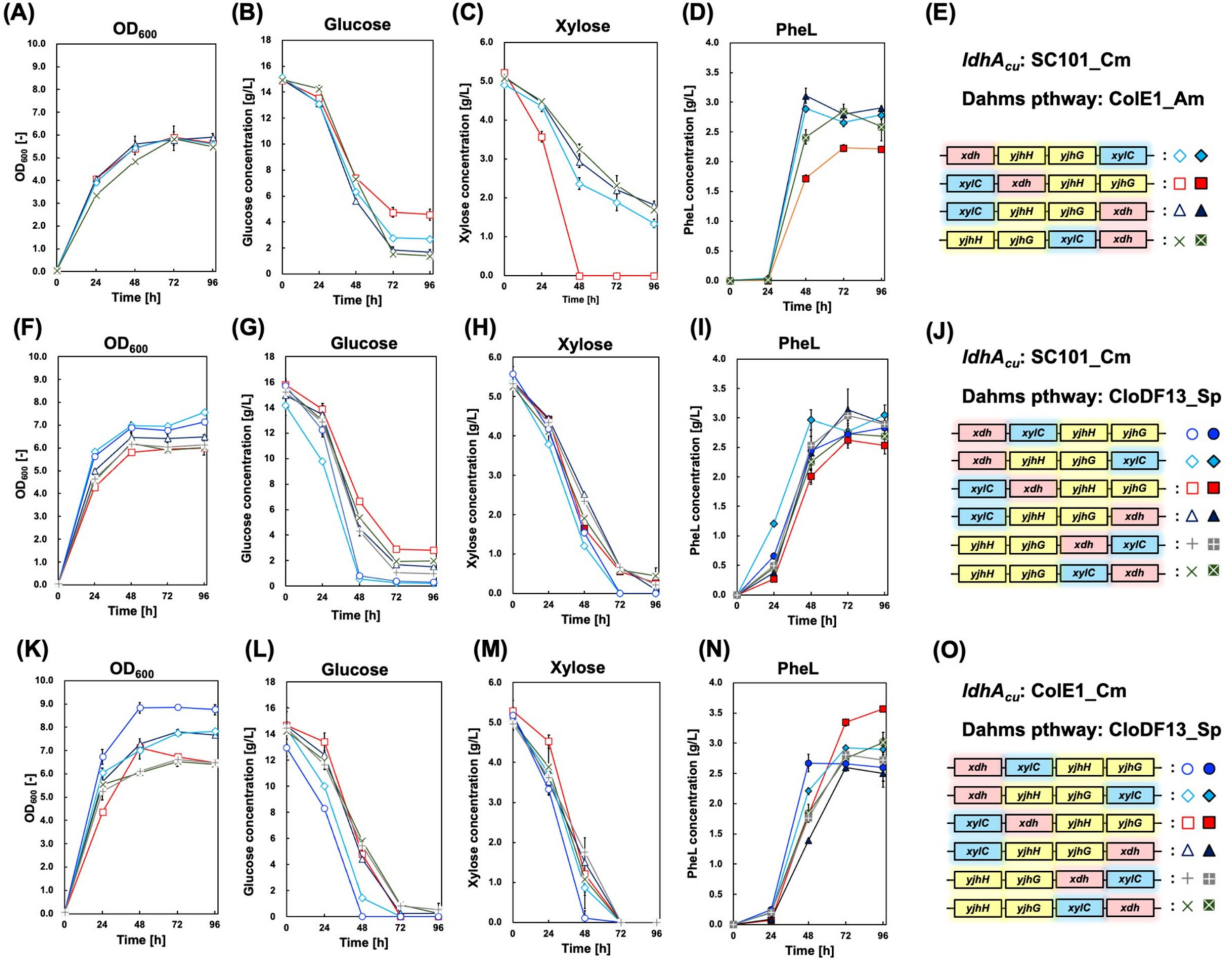
Culture profiles of PheL-producing strains expressing the Dahms pathway genes and *ldhA_Cu_*. Time-course profiles show (**A**) bacterial cell growth, (**B**) glucose consumption, (**C**) xylose consumption, and (**D**) PheL production. (**E**) The gene orders used in each plasmid construct are illustrated alongside corresponding symbols. Diamonds, squares, triangles, and crosses represent strains C40_pS_12dahms2, C40_pS_12dahms3, C40_pS_12dahms4, and C40_pS_12dahms6, respectively. Panels A through E show the results obtained from the PheL-producing strains carrying ColE1-based plasmids expressing Dahms pathway genes and pSAK-ldhA. Time-course profiles show (**F**) bacterial cell growth, (**G**) glucose concentration, (**H**) xylose concentration, and (**I**) PheL production. (**J**) The gene orders used in each plasmid construct are illustrated alongside corresponding symbols. Circles, diamonds, squares, triangles, daggers, and crosses represent strains C40_pS_CDdahms1, C40_pS_CDdahms2, C40_pS_CDdahms3, C40_pS_CDdahms4, C40_pS_CDdahms5, and C40_pS_CDdahms6, respectively. Panels F through J show the results obtained from the PheL-producing strains carrying CloDF13-based plasmids expressing Dahms pathway genes and pSAK-ldhA. Time-course profiles show (**K**) optical density at 600 nm (OD₆₀₀), (**L**) glucose concentration, (**M**) xylose concentration, and (**N**) PheL production over 72 h of cultivation in M9Y medium. (**O**) The gene orders used in each plasmid construct are illustrated alongside corresponding symbols. Circles, diamonds, squares, triangles, daggers, and crosses represent strains C40_pZCm_CDdahms1, C40_pZCm_CDdahms2, C40_pZCm_CDdahms3, C40_pZCm_CDdahms4, C40_pZCm_CDdahms5, and C40_pZCm_CDdahms6, respectively. Panels K through O show the results obtained from the PheL-producing strains carrying CloDF13-based plasmids expressing Dahms pathway genes and pZE12_Cm-ldhA. Data are presented as mean ± standard deviation of three biological replicates.

Four viable transformants were cultured in M9Y medium containing both glucose and xylose, and their culture profiles were analyzed (Fig. 7A through E). All strains, including C40_12pS, showed similar cell growth kinetics, reaching a maximum OD_600_ of approximately 6. However, no strain, including C40_12pS, completely consumed the additional glucose. Interestingly, only C40_12pS and C40_pS_12dahms3, which carried the *xylC–xdh–yjhHG* genes in tandem, completely assimilated xylose. Both plasmids shared a key feature: the placement of *xdh* and *xylC* upstream of *yjhHG*, emphasizing the importance of early steps in the Dahms pathway, especially the conversion of xylose to xylonate, in enabling xylose assimilation.

Despite complete xylose utilization, PheL titers in these two strains were limited to ~2.0 g/L. Conversely, the other three transformants, which only partially utilized xylose, produced nearly 3.0 g/L of PheL. Notably, strains that consumed the most glucose yielded the highest PheL titers. These results suggest that overexpression of the Dahms pathway genes does not linearly improve product formation. Instead, expression levels must be fine-tuned according to the target product, highlighting the importance of pathway balance over brute-force gene amplification.

### Customized expression system to produce PheL from a mixture of glucose and xylose

In the previous section, we demonstrated that gene order, which affects the expression balance within the Dahms pathway, plays a key role in PheL production from mixed sugars. Plasmids carrying the ColE1 replication origin were used to express the Dahms pathway gene cluster. Here, we investigated the impact of expression levels by replacing the ColE1 origin with the CloDF13 replication origin to modulate plasmid copy number.

Six *E. coli* transformants were generated, each harboring a CloDF13-based plasmid with the Dahms pathway genes (*xdh*, *xylC*, and *yjhHG*) arranged in one of six tandem permutations, as done previously with ColE1-based vectors. The strains were cultured in M9Y medium supplemented with glucose and xylose, and their culture profiles were analyzed (Fig. 7F through J). Two strains, C40_pS_CDdahms1 and C40_pS_CDdahms2, reached a maximum OD_600_ of approximately 7.0, higher than that observed with ColE1-based plasmids. Notably, both strains exhibited near-complete assimilation of additional glucose after 72 h of cultivation. Furthermore, they completely consumed the additional xylose; however, the PheL titer remained around 3.0 g/L similar to that of strains carrying ColE1-based plasmids.

All CloDF13-based transformants exhibited improved xylose assimilation, with residual xylose levels remaining below 1.0 g/L after 72 h (Fig. 7H), suggesting enhanced xylose utilization compared to that in ColE1-based systems (Fig. 7C vs. Fig. 7H). We also quantified the mRNA expression levels of four Dahms pathway genes (*xdh*, *xylC*, *yjhH*, and *yjhG*; Fig. S6). The expression levels aligned well with the gene order in each plasmid. While cross-plasmid comparisons of gene expression (Fig. S4) were inconclusive due to copy number variability, the intra-plasmid expression profiles were internally consistent and informative (Fig. S6).

Next, we focused on optimizing the expression of *ldhA_Cu_* for PheL production in *E. coli* strains carrying the Dahms pathway gene cluster. To this end, we reselected an expression plasmid from the previously constructed library of 24 combinations of replication origins and selection markers. Among them, pZE_Cm_ldhA was chosen and introduced into six *E. coli* strains already harboring the Dahms pathway gene cluster on pZCD_Sp. Among all transformants, the one carrying pZE_Am exhibited the highest yield of PheL from glucose. Nevertheless, its final PheLA titer and total glucose consumption were lower than those achieved by the transformants carrying pZE_Cm_ldhA and pZE_Sp_ldhA. We assumed that the low glucose consumption in the pZE_Am strain would be a critical limitation for future work, particularly large-scale production in a jar fermentor. Consequently, pZE_Cm_ldhA and pZE_Sp_ldhA were chosen as the most suitable plasmids for PheL production. Given that spectinomycin resistance was competitive with the plasmid encoding the Dahms pathway gene cluster, pZE_Cm_ldhA was finally adopted for the experiment involving PheL production from mixed glucose and xylose substrates. The resulting transformants were cultured in M9Y medium supplemented with glucose and xylose, and their culture profiles were examined (Fig. 7K through O). The maximum cell density in all strains exceeded an OD_600_ of 6.0, comparable to that achieved using pSAK-ldhA. A similar trend in sugar utilization was observed, especially in strains with *xdh* located upstream in the Dahms pathway cluster, which rapidly assimilated both glucose and xylose. All transformants carrying pZE_Cm_ldhA completely consumed the additional glucose, whereas glucose remained in the medium for four of the six strains carrying pSAK-ldhA.

Xylose uptake was slower in the pZE_Cm_ldhA strains than in their pSAK-ldhA counterparts. These findings suggest that the expression of a heterologous biosynthetic gene, such as *ldhA_Cu_*, can influence not only endogenous glucose metabolism but also the performance of the heterologously expressed Dahms pathway. The highest PheL production was observed in the C40_pZCm_CDdahms3 strain, which harbored *xylC*, *xdh*, and *yjhHG* arranged in tandem. This strain achieved a titer of 3.57 g/L PheL with the yield from glucose 0.24 g/g. The maximal PheL yield achieved using glucose and xylose (0.24 g/g) was lower than the yield obtained when using only glucose (0.34 g/g). We specifically concentrated on results from the strains carrying ColE1 origin derivative plasmids, as they alone demonstrated yields exceeding 0.30 g/g. Within that group, the strains with Am and Km resistance achieved PheL yields over 3.0 g/g; however, neither of these two strains fully assimilated the initially added glucose. This incomplete glucose assimilation likely accounts for the high PheL yield observed in these strains. The champion strain for mixed sugar assimilation, C40_pZCm_CDdahms3, fully consumed both the initially added glucose and xylose after 72 h of cultivation. Crucially, this strain achieved the highest production titer (3.57 g/L) under the condition of complete substrate utilization, representing the most robust performance for mixed-sugar fermentation in this study.

In this work, we separately regulated chemical production in the shikimate pathway and the driving of the TCA cycle by utilizing metabolic pathway compartmentalization and the Dahms pathway. A related study reported using a quorum sensing system as a strategy to initiate the production phase for shikimate-derived compounds (34). Integrating our system with such a dynamic regulation system could potentially lead to a more stringent separation between the production phase and the cell growth phase.

### Conclusion

In this study, we optimized the expression of a target gene to enhance PheL production by systematically altering key plasmid DNA components. By shuffling combinations of replication origins and antibiotic resistance markers, we observed significant differences in metabolic performance, including changes in PheL titer and yield. The highest yield from glucose, 0.34 g/g, and a titer of 5.45 g/L were achieved representing, to our knowledge, the highest reported yield of PheL under comparable batch conditions.

Comprehensive data were collected on plasmid selection and culture performance, enabling the development of a tailored framework for selecting suitable expression vectors for targeted compound production. To extend this approach to biomass-derived substrates, we further engineered a metabolic pathway-compartmentalized *E. coli* strain incorporating a Dahms pathway gene cluster to assimilate xylose. This platform enabled successful PheL production from co-substrates (glucose and xylose), achieving a titer of 3.57 g/L and a yield of 0.24 g/g from glucose. Overall, our findings highlight the importance of fine-tuning gene expression via plasmid architecture and gene order to match the metabolic demands of the host system. The resulting PheL-producing strains demonstrate promise for scalable bioproduction and further optimization of fermentation strategies.

## MATERIALS AND METHODS

### Strains and plasmid construction

The strains and plasmids used in this study are listed in Table S1. *E. coli* NovaBlue-competent cells (Novagen) were used for gene cloning. Polymerase chain reaction (PCR) was performed using KOD FX Neo DNA polymerase (Toyobo, Osaka, Japan), and the primer pairs are listed in Table S2. Each gene was assembled into the respective plasmid using Gibson Assembly (New England Biolabs, Ipswich, MA, USA). The plasmids used in this study were constructed as follows.

**pUC18-Ptrc** was constructed as follows: The gene fragment containing the *trc* promoter and the pUC18 backbone were amplified by PCR using pZE12-Ptrc or pUC18 as templates, with trc_to_puc_fw and trc_to_puc_rv, or inv_puc_fw and inv_puc_rv as the primer pairs. The amplified fragments were joined, and the resulting plasmid was named pUC18-Ptrc. **pZA23-Ptrc** was constructed as follows: The *trc* promoter and pZA23MCS were amplified by PCR using pZE12-Ptrc or pUC18 as templates, with trc_to_pza_fw and trc_to_pza_rv, or inv_pza_fw and inv_pza_rv as the primer pairs. The amplified fragments were assembled, and the resulting plasmid was designated pZA23-Ptrc. **pZCD-Ptrc** was constructed as follows: The *trc* promoter, CloDF13 origin, and spectinomycin resistance gene were amplified by PCR using pZE12-Ptrc and pCDFDuet-1 as templates, with trc_to_pzcd_fw and trc_to_pzcd_rv, or pzcd_ori_res_fw and pzcd_ori_res_rv as the primer pairs. The amplified fragments were assembled, and the resulting plasmid was named pZCD-Ptrc. **pUC18-ldhA** was constructed as follows: The gene fragment *ldhA* and the pUC18-Ptrc backbone were amplified by PCR using the synthetic gene *ldhA* or pUC18-Ptrc as templates, with ldhA_to_trc_fw and ldhA_to_trc_rv, or inv_trc_fw and inv_trc_rv as the primer pairs. The amplified fragments were assembled, and the resulting plasmid was named pUC18-ldhA. **pZE12-ldhA**, **pZA23-ldhA**, **pZCD-ldhA**, **pZC12-ldhA**, and **pSAK-ldhA** were created using appropriate templates and primer pairs according to a similar scheme. **pUC18_Km-ldhA** was constructed as follows: The gene fragment encoding kanamycin resistance and the pUC18-ldhA plasmid without ampicillin resistance were amplified by PCR using pZA23-Ptrc or pUC18-ldhA as templates, and km_to_puc_fw and km_to_puc_rv, or inv_puc_ldha_fw and inv_puc_ldha_rv as the primer pairs. The amplified fragments were assembled, and the resulting plasmid was named pUC18_Km-ldhA. **pUC18_Cm-ldhA** and **pUC18_Sp-ldhA** were constructed using appropriate templates and primer pairs, following the same strategy.

**pZE12_Km-ldhA** was constructed as follows: The gene fragment encoding kanamycin resistance and the pZE12-ldhA plasmid without ampicillin resistance were amplified by PCR using pZA23-Ptrc or pZE12-ldhA as templates, and km_to_pze_fw and km_to_pze_rv, or inv_pze_ldha_fw and inv_pze_ldha_rv as the primer pairs. The amplified fragments were assembled, and the resulting plasmid was named pZE12_Km-ldhA. **pZE12_Cm-ldhA** and **pZE12_Sp-ldhA** were constructed using appropriate templates and primer pairs, according to a similar scheme. **pZA23_Am-ldhA** was constructed as follows: The gene fragment encoding ampicillin resistance and the pZA23-ldhA plasmid without kanamycin resistance were amplified by PCR using pZE12-Ptrc or pZA23-ldhA as templates, and am_to_pza_fw and am_to_pza_rv, or inv_pza_ldha_fw and inv_pza_ldha_rv as the primer pairs. The amplified fragments were assembled, and the resulting plasmid was named pZA23_Am-ldhA. **pZA23_Cm-ldhA** and **pZA23_Sp-ldhA** were created using appropriate templates and primer pairs, following the same strategy.

**pZCD_Am-ldhA** was constructed as follows: The gene fragment encoding ampicillin resistance and the pZCD-ldhA plasmid lacking spectinomycin resistance were amplified by PCR using pZE12-Ptrc or pZCD-ldhA as templates, and am_to_pzcd_fw and am_to_pzcd_rv, or inv_pzcd_ldha_fw and inv_pzcd_ldha_rv as the primer pairs. The amplified fragments were assembled, and the resulting plasmid was named **pZCD_**Am-ldhA.

**pZCD_Km-ldhA** and **pZCD_Cm-ldhA** were constructed using appropriate templates and primer pairs, following the same strategy.

**pZC12_Am-ldhA** was constructed as follows: The gene fragment encoding ampicillin resistance and the pZC12-ldhA plasmid lacking spectinomycin resistance were amplified by PCR using pZE12-Ptrc or pZC12-ldhA as templates, with am_to_pzc12_fw and am_to_pzc12_rv, or inv_pzc12_ldha_fw and inv_pzc12_ldha_rv as the primer pairs. The amplified fragments were assembled, and the resulting plasmid was named pZC12_Am-ldhA. **pZC12_Km-ldhA** and **pZC12_Cm-ldhA** were constructed using appropriate templates and primer pairs, following the same strategy.

**pSAK_Am-ldhA** was constructed as follows: The gene fragment encoding ampicillin resistance and the pSAK-ldhA plasmid lacking chloramphenicol resistance were amplified by PCR using pZE12-Ptrc or pSAK-ldhA as templates, with am_to_psak_fw and am_to_psak_rv, or inv_psak_ldha_fw and inv_psak_ldha_rv as the primer pairs. The amplified fragments were assembled, and the resulting plasmid was named pSAK_Am-ldhA. **pSAK_Km-ldhA** and **pSAK_Sp-ldhA** were constructed using appropriate templates and primer pairs, following the same strategy.

pZE12-dahms2 was constructed as follows: The gene fragments *xdh*, *xylC*, and *yjhHG* were amplified by PCR using pZE12-x as the template and xdh_sali_fw/xdh_sali_rv, xylc_kpni_fw/xylc_kpni_rv, and yjhhg_hindiii_fw/yjhhg_hindiii_rv as the primer pairs. The fragments were inserted into the SalI, KpnI, or HindIII restriction sites of pZE12MCS, and the resulting plasmid was named pZE12-dahms2. Other ColE1-based plasmids expressing genes of the Dahms pathway listed in Table S1 were constructed using appropriate templates and primer pairs according to a similar strategy.

**pZCD-dahms1** was constructed as follows: The *trc* promoter cassette, including the Dahms pathway gene cluster, and the pZCD-Ptrc backbone lacking promoter and terminator regions were amplified by PCR using pZE12-x and pZCD-Ptrc as templates, with trc_pzcd_fw/trc_pzcd_rv and inv_pzcd_dahms_fw/inv_pzcd_dahms_rv as the primer pairs. The amplified fragments were assembled, and the resulting plasmid was named pZCD-dahms1. Other CloDF13-based plasmids expressing Dahms pathway genes (Table S1) were constructed using appropriate templates and primer pairs following a similar method. Plasmids were transformed into bacterial strains using a Gene Pulser II (Bio-Rad, Hercules, CA, USA). Where applicable, 100 µg·mL^−1^ ampicillin, 50 µg·mL^−1^ kanamycin, and 15 µg·mL^−1^ chloramphenicol were added to the media for selection. The transformants generated in this study are listed in Table S1. **pTF-**Δ**xylAB** was constructed as follows: Gene fragments for *xylAB* deletion were amplified by PCR using *E. coli* K-12 MG1655 genomic DNA as the template and the corresponding primer pairs (Table S2). The pTF plasmid fragment was amplified by PCR using pTargetF as the template and inv_ptf_fw/inv_ptf_rv as the primer pairs. The gene deletion fragments were assembled with the pTF backbone, and the resulting plasmid was named pTF-ΔxylAB. **pTF-**N20ΔxylAB was constructed as follows: To introduce the N20 sequence into pTF-ΔxylAB, plasmid fragments were amplified by PCR using pTF-ΔxylAB as the template and inv_n20_xylAB_fw/inv_n20_xylAB_rv as the primer pairs (Table S2). The resulting fragments were self-assembled, and the final plasmid was named pTF-N20ΔxylAB.

### Inactivation of chromosomal genes

To delete the chromosomal genes (*sdaB, tdcG*, and *xylAB*), a CRISPR-Cas9 two-plasmid system incorporating pTargetF and pCas was used (32). Each gene was inactivated using an appropriate plasmid derived from pTargetF. The strains and their phenotypes are summarized in Table S1.

### Culture conditions

M9Y medium was used for PheL production in 5 mL test tube-scale cultures. M9Y minimal medium contained (per liter): glucose, 20 g, or a mixture of 15 g glucose and 5 g xylose; yeast extract, 5 g; NaCl, 0.5 g; Na_2_HPO_4_‧12H_2_O, 17.1 g; KH_2_PO_4_, 3 g; NH_4_Cl, 2 g; MgSO_4_‧7H_2_O, 246 mg; CaCl_2_‧2H_2_O, 14.7 mg; FeSO_4_‧7H_2_O, 2.78 mg; thiamine hydrochloride, 10 mg; L-phenylalanine, 100 mg; L-tyrosine, 40 mg; and L-tryptophan, 40 mg (Phe, Tyr, and Trp were included because CFT3 is auxotrophic for these amino acids).

For culturing CFT3 derivative strains, 10 mM sodium pyruvate was added to support bacterial growth during the early phase. When necessary, ampicillin, kanamycin, or chloramphenicol was added to final concentrations of 100, 50, and 15 µg·mL⁻¹, respectively. Each pre-culture was seeded into 5 mL of M9Y medium at an initial OD₆₀₀ of 0.05 in a test tube. Cultures were incubated at 37°C in a shaker at 180 rpm. IPTG (0.1 mM) was added when cultures reached an OD₆₀₀ of 0.5.

### Analytical methods

Cell growth was monitored by measuring OD_600_ using a UVmini-1240 spectrophotometer (Shimadzu, Kyoto, Japan). Glucose concentrations in culture supernatants were measured using the Glucose CII test (Wako, Kyoto, Japan) according to the manufacturer’s instructions.

The amount of PheL produced was analyzed using high-performance liquid chromatography (HPLC; Shimadzu) equipped with a 5C18-AR-II column (Nacalai Tesque, Kyoto, Japan). Culture supernatants were separated by centrifugation at 21,880 × *g* for 5 min. The column was operated at 30°C with a flow rate of 1.2 mL·min^−1^. A dual-solvent system was used, consisting of solvent A (50 mM phosphate buffer, pH 2.5) and solvent B (acetonitrile).

The gradient was as follows: 80% A from 0–3 min, gradually shifted to 50:50 A:B from 3–6 min, maintained from 6–7 min, and returned to 80% A from 7–13 min. Product concentrations were determined by UV detection at 254 nm using an SPD-20AV detector (Shimadzu).

### Quantification of the transcriptional level of mRNA via real-time PCR

Total RNA was isolated from individual cultures using the NucleoSpin RNA Plus Kit (Takara Bio, Shiga, Japan), following the manufacturer’s instructions. Reverse transcription was performed using ReverTra Ace (TOYOBO), and the primer pairs are listed in Table S2. Quantitative real-time PCR was conducted using a LightCycler 96 (Roche Diagnostics, Mannheim, Germany) with THUNDERBIRD SYBR qPCR Mix (TOYOBO). The normalized transcriptional level of each mRNA was calculated by the relative quantification method, using the *mdoG* gene, which encodes glucan biosynthesis protein G as the housekeeping reference. The oligonucleotide primers used in this study are listed in Table S2.

### Clustering analysis of culture profiles

X-means consensus clustering was performed as previously described (34). The initial number of clusters was set to two (*k*_min_), and the algorithm was allowed to explore up to 20 (*k*_max_). X-means clustering was implemented using the NumPy (ver. 1.23.5), Pandas (ver. 2.3.0), Matplotlib (ver. 3.10.3), and Scikit-learn (ver. 1.7.0) libraries in Python.

## SUPPLEMENTAL MATERIAL

Supplemental material aem.02270-25-s0001.doc Figures S1 to S6, sequence of the synthetic *ldhA_Re* gene, and Tables S1 to S6.

ASM does not own the copyrights to Supplemental Material that may be linked to, or accessed through, an article. The authors have granted ASM a non-exclusive, world-wide license to publish the Supplemental Material files. Please contact the corresponding author directly for reuse.
