# Supplementary material for: Metabolic and plasmid engineering to produce D-phenyllactic acid from glucose–xylose co-substrates in Escherichia coli
Source: Appl Environ Microbiol. 2025 Dec 3;91(12):e02270-25. doi: 10.1128/aem.02270-25 (PMC12724218; doi:10.1128/aem.02270-25)
Supplement: Supplemental material — Figures S1 to S6, sequence of the synthetic ldhA_Re gene, and Tables S1 to S6. [file aem.02270-25-s0001.doc]

**Supplementary materials**

**Supplementary Figures**

**Figure S1**

**Figure S1 | Culture profiles of PheL-producing strains carrying ColE1 and pUC-based plasmids.** Time courses of (**A**) and (**B**) glucose consumption and OD_600_, and (**C**) and (**D**) PheL production. Circles, squares, diamonds and triangles indicate ampicillin, kanamycin, chloramphenicol and spectinomycin of the plasmids, respectively. Data are presented as the mean ± standard deviation of three independent experiments.

**Figure S2**

**Figure S2 | Culture profiles of PheL-producing strains carrying p15A and CloDF13-based plasmids.** Time courses of (**A**) and (**B**) glucose consumption and OD_600_, and (**C**) and (**D**) PheL production. Circles, squares, diamonds and triangles indicate ampicillin, kanamycin, chloramphenicol and spectinomycin of the plasmids, respectively. Data are presented as the mean ± standard deviation of three independent experiments.

**Figure S3**

**Figure S3 | Culture profiles of PheL-producing strains carrying ColA and SC101-based plasmids.** Time courses of (**A**) and (**B**) glucose consumption and OD_600_, and (**C**) and (**D**) PheL production. Circles, squares, diamonds and triangles indicate ampicillin, kanamycin, chloramphenicol and spectinomycin of the plasmids, respectively. Data are presented as the mean ± standard deviation of three independent experiments.


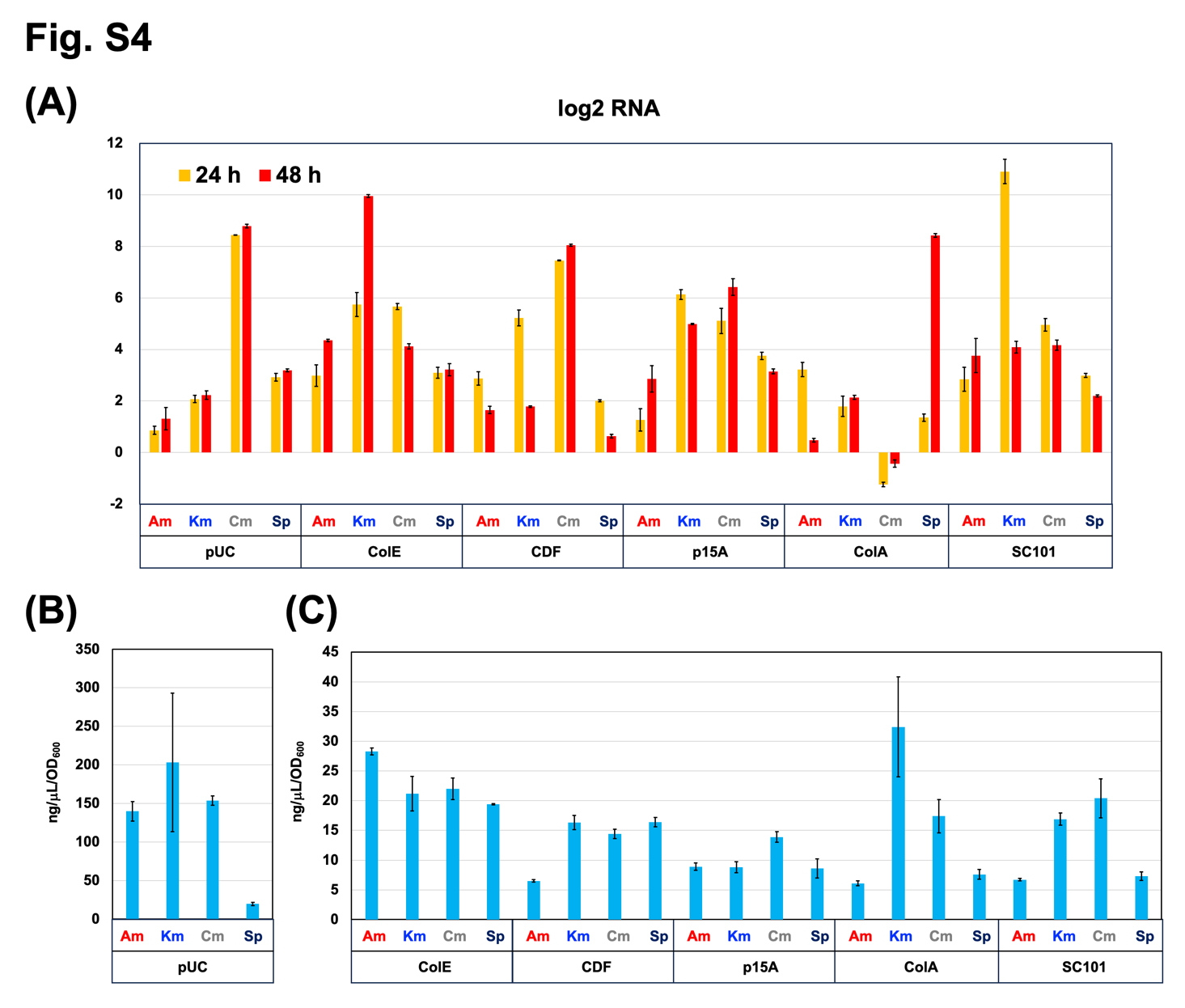
**Figure S4**

**Figure S4 | (A) The expression levels of mRNA of ldhA_Re after 24 h and 48 h cultivation, (B) and (C) the concentration of each plasmid per unit of bacterial cell after 18 h cultivation in 24 kinds of transformants.** Orange and red bars indicate the results after 24 h and 48 h, respectively. Data are presented as the mean ± standard deviation of three independent experiments.

**Figure S5**

**Figure S5 |Results for x-means clustering analysis.** The result of the culture profiles of OD_600_, glucose consumption and PheL production after 24 h and 72 h cultivation, yield of PheL from glucose after 72 h cultivation, and the expression levels of mRNA of ldhA_Re after 24 h and 48 h in 24 transformants carrying 24 kinds of different plasmids. Blue symbols include CFT3_pZE_Am, CFT3_pZE_Cm and CFT3_pZE_Sp, respectively. A sky blue symbol indicates CFT3_pZE_Km. Blue symbols include the other strains. The plots data was summarized in Table S6.

**Figure S6**

**Figure S6 | The expression levels of mRNAs concerning genes Dahms pathway after 48 h cultivation in transformants carrying pSAK-ldhA** **and CloDF13-based plasmids to express the genes concerning Dahms pathway.** Blue, orange, navy and gray bars indicate the results of *xdh*, *xylC*, *yjhH*, and *yjhG*, respectively. Data are presented as the mean ± standard deviation of three independent experiments.

**Supplementary Information**

Sequence of the synthetic *ldhA_Re* gene

CATCATCATCATGGTATGCCTGCACCGCAGATTCTGCAGGTTGGTCCGCTGGCACCGCGTACCAATGCAACCCTGCAGCAGCATTATGGTGCAGCAGCCCTGTGGCAGCAGGCAGATCCGATTGCATGGGCACGTAGCGAAGGTCAGCAGGTTCGTGTTGTTGTTACCAGCGCACGTCATGGTTGTAGCGCAGCACTGATTGATGCACTGCCTCGTCTGGAAGCAATTGTTAGCTTTGGTGTTGGTTATGATGCAATTGCCCTGGATGCAGCACGTGCCCGTGGTATTCAGGTTAGCAATACACCGGATGTTCTGAATGATTGTGTTGCAGATCTGGCATTTGGTCTGCTGCTGGACGCAGCGCGTGGTATTGCACATGGTGATCGTTTTGTTCGTGCAGGTCGTTGGCCTCAGGGTAGCTTTCCGCTGACCACCCGTGTTAGCGGTAAAAAACTGGGTATTCTGGGTCTGGGTCGTATTGGTGAAATTGTTGCACGTCGTGCACAGGGTTTTGATATGGAAATTGCCTATAATAACCGTCGTCCGCGTGAAGGTGCACCGTGGCGTTTTGAACCGGATCTGAAAGCACTGGCAACCTGGGCAGATTTTCTGGTTGTTGCAACCGTTGGTGGTCCGAGCACCGCAGGTCTGGTTAGCCGTGAAGTTATTGATGCCCTGGGTCCGCGTGGCATTCTGGTTAATGTGAGCCGTGGTAGCGTGATTGATGAAGCAGCAATGGTTGAAGCCCTGGTTGATGGTCGTCTGGGTGGTGCCGGTCTGGATGTTTTTCAGGATGAACCGAATGTTCCGCCTGCACTGATGGCACTGGATAATGTTGTTCTGGCTCCGCATATGGCAAGCGGCACCCATGAAACCCGTGCAGCAATGACCGCACTGACCCTGCAGAACCTGGATGCCTTTCTGGCAGATGGTCGTGTTCTGACACCGGTTCTGTAAGATCCGAGCTCGAGA

**Supplementary Tables**

**Table S1. Strains, plasmids, and transformants used in this study**

| Strain or plasmids | Genotype | Source or reference |
| --- | --- | --- |
| Strains |  |  |
| NovaBlue | endA1 hsdR17(rK12-mK12+) supE44 thi-I gyrA96 relA1 lac recA1/F’ [proAB+ lacIq ZΔM15::Tn10(Tetr)]; used for gene cloning | Novagen |
| ATCC 31882 | L-Phenylalanine-overproducing strain | American Type Culture Collection |
| CFT3 | ATCC31882 ptsHI::P_A1lacO-1_-*Glk*-*GalP*Δ*pykF*Δ*pykA* | [1] |
| CFT037 | ATCC31882 *ptsHI*::P_A1lacO-1_-*Glk*-*GalP*Δ*pykF*Δ*pykA*Δ*ppc*Δ*iclR*Δ*ppsA*Δ*pck*Δ*edd*Δ*sdaA* | [2] |
| CFT038 | CFT037Δ*sdaB* | This study |
| CFT039 | CFT038Δ*tdcG* | This study |
| CF'040 | CFT039ΔxylAB | This study |
| CFT3_pU_Am | CFT3 harboring pUC18-ldhA | This study |
| CFT3_pU_Km | CFT3 harboring pUC18_Km-ldhA | This study |
| CFT3_pU_Cm | CFT3 harboring pUC18_Cm-ldhA | This study |
| CFT3_pU_Sp | CFT3 harboring pUC18_Sp-ldhA | This study |
| CFT3_pZE_Am | CFT3 harboring pZE12-ldhA | This study |
| CFT3_pZE_Km | CFT3 harboring pZE12_Km-ldhA | This study |
| CFT3_pZE_Cm | CFT3 harboring pZE12_Cm-ldhA | This study |
| CFT3_pZE_Sp | CFT3 harboring pZE12_Sp-ldhA | This study |
| CFT3_pZA_Am | CFT3 harboring pZA23_Am-ldhA | This study |
| CFT3_pZA_Km | CFT3 harboring pZA23-ldhA | This study |
| CFT3_pZA_Cm | CFT3 harboring pZA23_Cm-ldhA | This study |
| CFT3_pZA_Sp | CFT3 harboring pZA23_Sp-ldhA | This study |
| CFT3_pCD_Am | CFT3 harboring pZCD_Am-ldhA | This study |
| CFT3_pCD_Km | CFT3 harboring pZCD_Km-ldhA | This study |
| CFT3_pCD_Cm | CFT3 harboring pZCD_Cm-ldhA | This study |
| CFT3_pCD_Sp | CFT3 harboring pZCD-ldhA | This study |
| CFT3_pZC_Am | CFT3 harboring pZC12_Am-ldhA | This study |
| CFT3_pZC_Km | CFT3 harboring pZC12_Km-ldhA | This study |
| CFT3_pZC_Cm | CFT3 harboring pZC12_Cm-ldhA | This study |
| CFT3_pZC_Sp | CFT3 harboring pZC12-ldhA | This study |
| CFT3_pSA_Am | CFT3 harboring pSAK_Am-ldhA | This study |
| CFT3_pSA_Km | CFT3 harboring pSAK_Km-ldhA | This study |
| CFT3_pSA_Cm | CFT3 harboring pSAK-ldhA | This study |
| CFT3_pSA_Sp | CFT3 harboring pSAK_Sp-ldhA | This study |
| C37_12_pS | CFT037 harboring pZE12MCS and pSAK-ldhA | This study |
| C37_12x_pS | CFT037 harboring pZE12-x and pSAK-ldhA | This study |
| C40_12x_pS | CFT040 harboring pZE12-x and pSAK-ldhA | This study |
| C40_pS_12dahms2 | CFT040 harboring pSAK-ldhA and pZE12-dahms2 | This study |
| C40_pS_12dahms3 | CFT040 harboring pSAK-ldhA and pZE12-dahms3 | This study |
| C40_pS_12dahms4 | CFT040 harboring pSAK-ldhA and pZE12-dahms4 | This study |
| C40_pS_12dahms6 | CFT040 harboring pSAK-ldhA and pZE12-dahms6 | This study |
| C40_pS_CDdahms1 | CFT040 harboring pSAK-ldhA and pZCD-dahms1 | This study |
| C40_pS_CDdahms2 | CFT040 harboring pSAK-ldhA and pZCD-dahms2 | This study |
| C40_pS_CDdahms3 | CFT040 harboring pSAK-ldhA and pZCD-dahms3 | This study |
| C40_pS_CDdahms4 | CFT040 harboring pSAK-ldhA and pZCD-dahms4 | This study |
| C40_pS_CDdahms5 | CFT040 harboring pSAK-ldhA and pZCD-dahms5 | This study |
| C40_pS_CDdahms6 | CFT040 harboring pSAK-ldhA and pZCD-dahms6 | This study |
| C40_pZCm_CDdahms1 | CFT040 harboring pZE12_Cm-ldhA and pZCD-dahms1 | This study |
| C40_pZCm_CDdahms2 | CFT040 harboring pZE12_Cm-ldhA and pZCD-dahms2 | This study |
| C40_pZCm_CDdahms3 | CFT040 harboring pZE12_Cm-ldhA and pZCD-dahms3 | This study |
| C40_pZCm_CDdahms4 | CFT040 harboring pZE12_Cm-ldhA and pZCD-dahms4 | This study |
| C40_pZCm_CDdahms5 | CFT040 harboring pZE12_Cm-ldhA and pZCD-dahms5 | This study |
| C40_pZCm_CDdahms6 | CFT040 harboring pZE12_Cm-ldhA and pZCD-dahms6 | This study |
| Plasmids |  |  |
| pUC18 | pUC ori, Am^r^ | Takara |
| pCDFDuet-1 | *P_T7_*, CloDF13 ori, Sp^r^ | Novagen |
| pZA23MCS | *P_AlacO-1_*, p15A ori, Km^r^ | Expressys |
| pTargetF | sgRNA sequence, pMB1 ori, Sp^r^ | Addgene |
| pCas | lambda Red recombinase, cas9 from *Streptococcus pyogenes*  MGAS5005, Km^r^ | Addgene |
| pZE12MCS | *P_trc_*, colE1 ori, Amp^r^ |  |
| pZE12-Ptrc | *P_trc_*, colE1 ori, Amp^r^ | [2] |
| pSAK-Ptrc | *P_trc_*, SC101 ori, Cm^r^ | [3] |
| pZC12Sp-Ptrc | *P_trc_*, ColA ori, Sp^r^ | [3] |
| pZE12-x | pZE12MCS containing *xdh* and *xylC* from *Caulobacter crescentus*,  and *yjhHG* from *E. coli* MG1655 in tandem | [4] |
| pTF-N20Δ*sdaB* | pTargetF containing sgRNA with donor editing template DNA to inactivate sdaB | [2] |
| pTF-N20Δ*tdcG* | pTargetF containing sgRNA with donor editing template DNA to inactivate tdcG | [2] |
| pUC18-Ptrc | *P_trc_*, pUC ori, Am^r^ | This study |
| pZA23-Ptrc | *P_trc_,* p15A ori, Km^r^ | This study |
| pZCD-Ptrc | *P_trc_*, CloDF13 ori, Sp^r^ | This study |
| pUC18-ldhA | pUC18-Ptrc containing ldhA from *Ralstonia eutropha* | This study |
| pZE12-ldhA | pZE12-Ptrc containing ldhA from *R. eutropha* | This study |
| pZA23-ldhA | pZA23-Ptrc containing ldhA from *R. eutropha* | This study |
| pZCD-ldhA | pZCD-Ptrc containing ldhA from *R. eutropha* | This study |
| pZC12-ldhA | pZC12Sp-Ptrc containing ldhA from *R. eutropha* | This study |
| pSAK-ldhA | pSAK-Ptrc containing ldhA from *R. eutropha* | This study |
| pUC18_Km-ldhA | pUC18-ldhA derivative, Km^r^ | This study |
| pUC18_Cm-ldhA | pUC18-ldhA derivative, Cm^r^ | This study |
| pUC18_Sp-ldhA | pUC18-ldhA derivative, Sm^r^ | This study |
| pZE12_Km-ldhA | pZE12-ldhA derivative, Km^r^ | This study |
| pZE12_Cm-ldhA | pZE12-ldhA derivative, Cm^r^ | This study |
| pZE12_Sp-ldhA | pZE12-ldhA derivative, Sp^r^ | This study |
| pZA23_Am-ldhA | pZA23-ldhA derivative, Am^r^ | This study |
| pZA23_Cm-ldhA | pZA23-ldhA derivative, Cm^r^ | This study |
| pZA23_Sp-ldhA | pZA23-ldhA derivative, Sp^r^ | This study |
| pZCD_Am-ldhA | pZCD-ldhA derivative, Am^r^ | This study |
| pZCD_Km-ldhA | pZCD-ldhA derivative, Km^r^ | This study |
| pZCD_Cm-ldhA | pZCD-ldhA derivative, Cm^r^ | This study |
| pZC12_Am-ldhA | pZC12-ldhA derivative, Am^r^ | This study |
| pZC12_Km-ldhA | pZC12-ldhA derivative, Km^r^ | This study |
| pZC12_Cm-ldhA | pZC12-ldhA derivative, Cm^r^ | This study |
| pSAK_Am-ldhA | pSAK-ldhA derivative, Am^r^ | This study |
| pSAK_Km-ldhA | pSAK-ldhA derivative, Km^r^ | This study |
| pSAK_Sp-ldhA | pSAK-ldhA derivative, Sp^r^ | This study |
| pZE12-dahms2 | pZE12MCS containing *xdh*, *yjhHG* and *xylC* in tandem | This study |
| pZE12-dahms3 | pZE12MCS containing *xylC*, *xdh* and *yjhHG* in tandem | This study |
| pZE12-dahms4 | pZE12MCS containing *xylC*, *yjhHG* and *xdh* in tandem | This study |
| pZE12-dahms5 | pZE12MCS containing *yjhHG*, *xdh* and *xylC* in tandem | This study |
| pZE12-dahms6 | pZE12MCS containing *yjhHG*, *xylC* and *xdh* in tandem | This study |
| pZCD-dahms1 | pZCD-Ptrc containing *xdh*, *xylC* and *yjhHG* in tandem | This study |
| pZCD-dahms2 | pZCD-Ptrc containing *xdh*, *yjhHG* and *xylC* in tandem | This study |
| pZCD-dahms3 | pZCD-Ptrc containing *xylC*, *xdh* and *yjhHG* in tandem | This study |
| pZCD-dahms4 | pZCD-Ptrc containing *xylC*, *yjhHG* and *xdh* in tandem | This study |
| pZCD-dahms5 | pZCD-Ptrc containing *yjhHG*, *xdh* and *xylC* in tandem | This study |
| pZCD-dahms6 | pZCD-PtrcS containing *yjhHG*, *xylC* and *xdh* in tandem | This study |
| pTF-Δ*xylAB* | pTargetF containing sgRNA with donor editing template DNA  to inactivate *xylAB* | This study |
| pTF-N20Δ*xylAB* | pTF-Δ*xylAB* with a universal N20 sequence | This study |

Table S2. Sequences of the oligonucleotide primers used in this study

| Oligonucleotide primers | Sequence |  |
| --- | --- | --- |
|  |
| trc_to_puc_fw | 5'- GGGCTGGCTTAACTATGTTGACAATTAATCATCCG -3' |  |
| trc_to_puc_rv | 5'- TTAGGCACCCCAGGCAAGGCCCAGTCTTTCGACTG -3' |  |
| inv_puc_fw | 5'- GCCTGGGGTGCCTAATGAGTGAGCT -3' |  |
| inv_puc_rv | 5'- TAGTTAAGCCAGCCCCGACACCCGC -3' |  |
| trc_to_pza_fw | 5'- TATCACGAGGCCCTTTCGTCTGTTGACAATTAATC -3' |  |
| trc_to_pza_rv | 5'- TGAGCGAGGAAGCGGAATAAAGGCCCAGTCTTTCG -3' |  |
| inv_pza_fw | 5'- TATTCCGCTTCCTCGCTCAC -3' |  |
| inv_pza_rv | 5'- GACGAAAGGGCCTCGTGATA -3' |  |
| trc_to_pzcd_fw | 5'- GAAGGCTCTCAAGGGAGAGTTTGTAGAAACGCAAAAAGGC -3' |  |
| trc_to_pzcd_rv | 5'- TCTCAAATGCCTGAGGTTTGACAGCTTATCATCGACTGC -3' |  |
| pzcd_ori_res_fw | 5'- CTCAGGCATTTGAGAAGCACACGG -3' |  |
| pzcd_ori_res_rv | 5'- CCCTTGAGAGCCTTCAACCCAGTC -3' |  |
| ldhA_to_trc_fw | 5'- CATCATCATCATGGTATGCCTGCACCGCAG -3' |  |
| ldhA_to_trc_rv | 5'- TCTCGAGCTCGGATCTTACAGAACCGGTGT -3' |  |
| inv_trc_fw | 5'- GATCCGAGCTCGAGATCTGC -3' |  |
| inv_trc_rv | 5'- GGTTTATTCCTCCTTATTTA -3' |  |
| km_to_puc_fw | 5'- GACGCTCAGTGGAACGAGCTCTCGAACCCCAGAGT -3' |  |
| km_to_puc_rv | 5'- GTGGCACTTTTCGGGCGGAATTGCCAGCTGGGGCG -3' |  |
| inv_puc_ldha_fw | 5'- CCCGAAAAGTGCCACCTGACGTCTA -3' |  |
| inv_puc_ldha_rv | 5'- GTTCCACTGAGCGTCAGACCCCGTA -3' |  |
| cm_to_puc_fw | 5'- GACGCTCAGTGGAACGTGAAGACGAAAGGGCCTCG -3' |  |
| cm_to_puc_rv | 5'- GTGGCACTTTTCGGGGAGCTCGATATCAAATTACG -3' |  |
| sp_to_puc_fw | 5'- GACGCTCAGTGGAACGGGATGATAAGTTTATCACC -3' |  |
| sp_to_puc_rv | 5'- GTGGCACTTTTCGGGCACCTAGATCCTTTACGCGT -3' |  |
| km_to_pze_fw | 5'- GGATTTTGGTCATGACGGAATTGCCAGCTGGGGCG -3' |  |
| km_to_pze_frv | 5'- TGGTTTCTTAGACGTGAGCTCTCGAACCCCAGAGT -3' |  |
| inv_pze_ldha_fw | 5'- ACGTCTAAGAAACCATTATT -3' |  |
| inv_pze_ldha_rv | 5'- TCATGACCAAAATCCCTTAA -3' |  |
| cm_to_pze_fw | 5'- GGATTTTGGTCATGAGAGCTCGATATCAAATTACG -3' |  |
| cm_to_pze_rv | 5'- TGGTTTCTTAGACGTGTGAAGACGAAAGGGCCTCG -3' |  |
| sp_to_pze_fw | 5'- GGATTTTGGTCATGAGGGATGATAAGTTTATCACC -3' |  |
| sp_to_pze_rv | 5'- TGGTTTCTTAGACGTCACCTAGATCCTTTACGCGT -3' |  |
| am_to_pza_fw | 5'- CGATATAAGTTGTTAGTCAGGTGGCACTTTTCGGG -3' |  |
| am_to_pza_rv | 5'- TGGTTTCTTAGACGTACTAGTGCTTGGATTCTCAC -3' |  |
| inv_pza_ldha_fw | 5'- ACGTCTAAGAAACCATTATT -3' |  |
| inv_pza_ldha_rv | 5'- TAACAACTTATATCGTATGG -3' |  |
| cm_to_pza_fw | 5'- CGATATAAGTTGTTAGAGCTCGATATCAAATTACG -3' |  |
| cm_to_pza_rv | 5'- TGGTTTCTTAGACGTGTGAAGACGAAAGGGCCTCG -3' |  |
| sp_to_pza_fw | 5'- CGATATAAGTTGTTACACCTAGATCCTTTACGCGT -3' |  |
| sp_to_pza_rv | 5'- TGGTTTCTTAGACGTGGGATGATAAGTTTATCACC -3' |  |
| am_to_pzcd_fw | 5'- CGAGTGAGCTAGCTAGTCAGGTGGCACTTTTCGGG -3' |  |
| am_to_pzcd_rv | 5'- ACGAATTGTTAGACAACTAGTGCTTGGATTCTCAC -3' |  |
| inv_pzcd_ldha_fw | 5'- TGTCTAACAATTCGTTCAAGCCGAG -3' |  |
| inv_pzcd_ldha_rv | 5'- TAGCTAGCTCACTCGGTCGCTACGC -3' |  |
| km_to_pzcd_fw | 5'- CGAGTGAGCTAGCTACGGAATTGCCAGCTGGGGCG -3' |  |
| km_to_pzcd_rv | 5'- ACGAATTGTTAGACAGAGCTCTCGAACCCCAGAGT -3' |  |
| cm_to_pzcd_fw | 5'- CGAGTGAGCTAGCTAGAGCTCGATATCAAATTACG -3' |  |
| cm_to_pzcd_rv | 5'- ACGAATTGTTAGACAGTGAAGACGAAAGGGCCTCG -3' |  |
| am_to_pzc12_fw | 5'- AAAACGAAAGGCTCAGTCAGGTGGCACTTTTCGGG -3' |  |
| am_to_pzc12_rv | 5'- GCCTCGTGATACGCCACTAGTGCTTGGATTCTCAC -3' |  |
| inv_pzc12_ldha_fw | 5'- GGCGTATCACGAGGCCCTTTCGTCT -3' |  |
| inv_pzc12_ldha_rv | 5'- TGAGCCTTTCGTTTTATTTGATGCC -3' |  |
| km_to_pzc12_fw | 5'- AAAACGAAAGGCTCACGGAATTGCCAGCTGGGGCG -3' |  |
| km_to_pzc12_rv | 5'- GCCTCGTGATACGCCGAGCTCTCGAACCCCAGAGT -3' |  |
| cm_to_pzc12_fw | 5'- AAAACGAAAGGCTCAGAGCTCGATATCAAATTACG -3' |  |
| cm_to_pzc12_rv | 5'- GCCTCGTGATACGCCGTGAAGACGAAAGGGCCTCG -3' |  |
| am_to_psak_fw | 5'- TATCAACAGGAGTCCGTCAGGTGGCACTTTTCGGG -3' |  |
| am_to_psak_rv | 5'- CCCTTTCGTCTTCACACTAGTGCTTGGATTCTCAC -3' |  |
| inv_psak_ldha_fw | 5'- GTGAAGACGAAAGGGCCTCG -3' |  |
| inv_psak_ldha_rv | 5'- CGCTTGGACTCCTGTTGATA -3' |  |
| km_to_psak_fw | 5'- TATCAACAGGAGTCCCGGAATTGCCAGCTGGGGCG -3' |  |
| km_to_psak_rv | 5'- CCCTTTCGTCTTCACGAGCTCTCGAACCCCAGAGT -3' |  |
| sp_to_psak_fw | 5'- TATCAACAGGAGTCCGAGCTCGATATCAAATTACG -3' |  |
| sp_to_psak_rv | 5'- CCCTTTCGTCTTCACGTGAAGACGAAAGGGCCTCG -3' |  |
| xdh_kpni_fw | 5'- AGAGGAGAAAGGTACATGAGCAGCGCCATTTATCC -3' |  |
| xdh_kpni_rv | 5'- GGGGGGGCCCGGTACTCATTAACGCCAACCTGCAT -3' |  |
| xylc_kpni_fw | 5'- AGAGGAGAAAGGTACATGACCGCACAGGTTACCTG -3' |  |
| xylc_kpni_rv | 5'- GGGGGGGCCCGGTACTCATTAAACCAGACGAACTT -3' |  |
| yjhhg_kpni_fw | 5'- AGAGGAGAAAGGTACATGAAAAAATTCAGCGGCAT -3' |  |
| yjhhg_kpni_fw | 5'- GGGGGGGCCCGGTACTTATCAGTTTTTATTCATAA -3' |  |
| xdh_sali_fw | 5'- GGCCCCCCCTCGAGGTAAAGAGGAGAAAGGATGAGCAGCGCCATTTATCC -3' |  |
| xdh_sali_rv | 5'- AGCTTATCGATACCGCTCGAGTCATTAACGCCAACCTGCAT -3' |  |
| xylc_sali_fw | 5'- GGCCCCCCCTCGAGGTAAAGAGGAGAAAGGATGACCGCACAGGTTACCTG -3' |  |
| xylc_sali_fw | 5'- AGCTTATCGATACCGCTCGAGTCATTAAACCAGACGAACTT -3' |  |
| yjhhg_sali_fw | 5'- GGCCCCCCCTCGAGGTAAAGAGGAGAAAGGATGAAAAAATTCAGCGGCAT -3' |  |
| yjhhg_sali_rv | 5'- AGCTTATCGATACCGCTCGAGTTATCAGTTTTTATTCATAA -3' |  |
| xdh_hindiii_fw | 5'- CGAGCGGTATCGATATAAAGAGGAGAAAGGATGAGCAGCGCCATTTATCC -3' |  |
| xdh_hindiii_rv | 5'- AGGAATTCGATATCATCATTAACGCCAACCTGCAT -3' |  |
| xylc_hindiii_fw | 5'- CGAGCGGTATCGATATAAAGAGGAGAAAGGATGACCGCACAGGTTACCTG -3' |  |
| xylc_hindiii_rv | 5'- AGGAATTCGATATCATCATTAAACCAGACGAACTT -3' |  |
| yjhhg_hindiii_fw | 5'- CGAGCGGTATCGATATAAAGAGGAGAAAGGATGAAAAAATTCAGCGGCAT -3' |  |
| yjhhg_hindiii_rv | 5'- AGGAATTCGATATCATTATCAGTTTTTATTCATAA -3' |  |
| inv_pzcd_dahms_fw | 5'- CTCAGGCATTTGAGAAGCACACGG -3' |  |
| inv_pzcd_dahms_rv | 5'- CCCTTGAGAGCCTTCAACCCAGTC -3' |  |
| trc_pzcd_fw | 5'- GAAGGCTCTCAAGGGAGAGTTTGTAGAAACGCAAAAAGGC -3' |  |
| trc_pzcd_rv | 5'- TCTCAAATGCCTGAGGTTTGACAGCTTATCATCGACTGC -3' |  |
| inv_ptf_fw | 5'- TAGATCTATTACCCTGTTAT -3' |  |
| inv_ptf_rv | 5'- TCTGCAGGTCGACTCTAGAG -3' |  |
| xylab_l600_fw | 5'- GAGTCGACCTGCAGAATGCAAGCCTATTTTGACCA -3' |  |
| xylab_l600_rv | 5'- CACAGACATTAAATGGGTATTTAACAGCGTTTCGT -3' |  |
| xylab_r600_fw | 5'- CATTTAATGTCTGTGATGCT -3' |  |
| xylab_r600_rv | 5'- AGGGTAATAGATCTATTACGCCATTAATGGCAGAA -3' |  |
| inv_n20_xylab_fw | 5'- TCAGGAACATCATTTGTCTCGTTTTAGAGCTAGAAATAGC -3' |  |
| inv_n20_xylab_rv | 5'- AAATGATGTTCCTGATAGTATTATACCTAGGACTG -3' |  |
| qrt_mdog_fw | 5'- TTGCACGACTCTAACGGTCT -3' |  |
| qrt_mdog_fw | 5'- TTCCATGGAGAAGCTGCTGA -3' |  |
| qrt_ldha_fw | 5'- TGAAGCAGCAATGGTTGAAG -3' |  |
| qrt_ldha_rv | 5'- ATATGCGGAGCCAGAACAAC -3' |  |

**Table S3. The values of PC1 and PC2 for the results in Figure 3(A)**

|  | PC1 | PC2 |
| --- | --- | --- |
| CFT3_pU_Am | -1.01E+00 | -9.26E-01 |
| CFT3_pU_Km | -5.75E-01 | -5.87E-01 |
| CFT3_pU_Cm | 1.71E+00 | 1.84E+00 |
| CFT3_pU_Sp | -2.72E-01 | -2.32E-01 |
| CFT3_pZE_Am | -2.49E-01 | 1.99E-01 |
| CFT3_pZE_Km | 7.41E-01 | 2.27E+00 |
| CFT3_pZE_Cm | 7.13E-01 | 1.14E-01 |
| CFT3_pZE_Sp | -2.08E-01 | -2.22E-01 |
| CFT3_pZA_Am | -2.90E-01 | -8.01E-01 |
| CFT3_pZA_Km | 5.54E-01 | -7.53E-01 |
| CFT3_pZA_Cm | 1.36E+00 | 1.57E+00 |
| CFT3_pZA_Sp | -5.99E-01 | -1.18E+00 |
| CFT3_pCD_Am | -8.66E-01 | -3.56E-01 |
| CFT3_pCD_Km | 8.81E-01 | 4.36E-01 |
| CFT3_pCD_Cm | 5.15E-01 | 9.69E-01 |
| CFT3_pCD_Sp | 2.62E-02 | -2.47E-01 |
| CFT3_pZC_Am | -1.65E+00 | -1.24E+00 |
| CFT3_pZC_Km | -6.78E-01 | -6.20E-01 |
| CFT3_pZC_Cm | -1.77E+00 | -1.57E+00 |
| CFT3_pZC_Sp | -8.33E-01 | 1.71E+00 |
| CFT3_pSA_Am | -3.02E-01 | -1.83E-02 |
| CFT3_pSA_Km | 2.59E+00 | 1.03E-01 |
| CFT3_pSA_Cm | 4.60E-01 | 1.30E-01 |
| CFT3_pSA_Sp | -2.47E-01 | -5.99E-01 |

**Table S4. The values of PC1 and PC2 for the results in Figure 3(B)**

|  | PC1 | PC2 |
| --- | --- | --- |
| CFT3_pU_Am | -8.06E-01 | -1.25E+00 |
| CFT3_pU_Km | -5.96E-01 | -5.41E-01 |
| CFT3_pU_Cm | -7.51E-01 | -7.69E-01 |
| CFT3_pU_Sp | -7.07E-01 | -4.27E-01 |
| CFT3_pZE_Am | -4.85E-01 | 1.13E+00 |
| CFT3_pZE_Km | -4.07E-01 | 7.68E-02 |
| CFT3_pZE_Cm | -3.51E-01 | 1.46E+00 |
| CFT3_pZE_Sp | -2.85E-01 | 1.79E+00 |
| CFT3_pZA_Am | -1.04E+00 | -1.45E+00 |
| CFT3_pZA_Km | -4.29E-01 | 1.43E-01 |
| CFT3_pZA_Cm | -6.84E-01 | 4.19E-01 |
| CFT3_pZA_Sp | -8.51E-01 | -8.35E-01 |
| CFT3_pCD_Am | 1.47E+00 | 1.06E+00 |
| CFT3_pCD_Km | -3.29E-01 | -2.32E+00 |
| CFT3_pCD_Cm | -6.29E-02 | -9.78E-01 |
| CFT3_pCD_Sp | 7.92E-01 | 8.63E-02 |
| CFT3_pZC_Am | 1.86E+00 | 7.92E-04 |
| CFT3_pZC_Km | -8.29E-01 | -9.97E-01 |
| CFT3_pZC_Cm | -8.06E-01 | 1.62E-01 |
| CFT3_pZC_Sp | -8.62E-01 | -1.99E-01 |
| CFT3_pSA_Am | 1.00E+00 | 5.04E-01 |
| CFT3_pSA_Km | 1.19E+00 | 1.21E+00 |
| CFT3_pSA_Cm | 2.26E+00 | 9.70E-01 |
| CFT3_pSA_Sp | 1.71E+00 | 7.51E-01 |

**Table S5. The benchmark for PheL production in the experiments using batch culture**

| Strains | production (g/L) | Yield (g/g) | Reference |  |
| --- | --- | --- | --- | --- |
|  |
| CFT3_pZE_Am | 4.76 | 0.37 | This study |  |
| CFT3_pZE_Km | 3.65 | 0.36 | This study |  |
| CFT3_pZE_Cm | 5.11 | 0.31 | This study |  |
| CFT3_pSA_Km | 4.84 | 0.27 | This study |  |
| CFT3_pSA_Sp | 4.36 | 0.28 | This study |  |
| C40_pZCm_CDdahms3 | 3.57^a^ | 0.24 | This study |  |
| MG-P10 | 1.42 | 0.035 | [5] |  |
| PHE02(P_cueR_::P37) | 5.4 | 0.12 | [6] |  |
| M-PAR-120 derivative strain | 2.2 | 0.22 | [7] |  |
| NST37/pMGA1/pHSGyiaE | 2.9 | 0.087 | [8] |  |

^a^ Co-substrates of glucose and xylose were used

**Table S6. The values of PC1 and PC2 for the results in Figure S5**

|  | PC1 | PC2 |
| --- | --- | --- |
| CFT3_pU_Am | -8.06E-01 | -1.25E+00 |
| CFT3_pU_Km | -5.96E-01 | -5.41E-01 |
| CFT3_pU_Cm | -7.51E-01 | -7.69E-01 |
| CFT3_pU_Sp | -7.07E-01 | -4.27E-01 |
| CFT3_pZE_Am | -4.85E-01 | 1.13E+00 |
| CFT3_pZE_Km | -4.07E-01 | 7.68E-02 |
| CFT3_pZE_Cm | -3.51E-01 | 1.46E+00 |
| CFT3_pZE_Sp | -2.85E-01 | 1.79E+00 |
| CFT3_pZA_Am | -1.04E+00 | -1.45E+00 |
| CFT3_pZA_Km | -4.29E-01 | 1.43E-01 |
| CFT3_pZA_Cm | -6.84E-01 | 4.19E-01 |
| CFT3_pZA_Sp | -8.51E-01 | -8.35E-01 |
| CFT3_pCD_Am | 1.47E+00 | 1.06E+00 |
| CFT3_pCD_Km | -3.29E-01 | -2.32E+00 |
| CFT3_pCD_Cm | -6.29E-02 | -9.78E-01 |
| CFT3_pCD_Sp | 7.92E-01 | 8.63E-02 |
| CFT3_pZC_Am | 1.86E+00 | 7.92E-04 |
| CFT3_pZC_Km | -8.29E-01 | -9.97E-01 |
| CFT3_pZC_Cm | -8.06E-01 | 1.62E-01 |
| CFT3_pZC_Sp | -8.62E-01 | -1.99E-01 |
| CFT3_pSA_Am | 1.00E+00 | 5.04E-01 |
| CFT3_pSA_Km | 1.19E+00 | 1.21E+00 |
| CFT3_pSA_Cm | 2.26E+00 | 9.70E-01 |
| CFT3_pSA_Sp | 1.71E+00 | 7.51E-01 |

**References**

1. Noda S, Shirai T, Oyama S, Kondo A. 2016. Metabolic design of a platform *Escherichia coli* strain producing various chorismate derivatives. Metab. Eng. 33, 119-129. doi: 10.1016/j.ymben.2015.11.007.
2. Noda S, Mori Y, Fujiwara R, Shirai T, Tanaka T, Kondo A., 2021. Reprogramming *Escherichia coli* pyruvate-forming reaction towards chorismate derivatives production. Metab. Eng. 67, 1-10. doi: 10.1016/j.ymben.2021.05.005.
3. Mori Y, Noda S, Shirai T, Kondo A. 2021. Direct 1,3-butadiene biosynthesis in *Escherichia coli* via a tailored ferulic acid decarboxylase mutant. Nat. Commun. 12, 2195-2206. doi: 10.1038/s41467-021-22504-6.
4. Fujiwara R, Noda S, Tanaka T, Kondo A., 2020. Metabolic engineering of Escherichia coli for shikimate pathway derivative production from glucose-xylose co-substrate. Nat Commun. 11, 279-290. doi: 10.1038/s41467-019-14024-1.
5. Wu W, Chen M, Li C, Zhong J, Xie R, Pan Z, Lin J, Qi F. 2024. Efficient production of phenyllactic acid in *Escherichia coli* via metabolic engineering and fermentation optimization strategies. Front. Microbiol. 15, 1457628. doi: 10.3389/fmicb.2024.1457628.
6. Zhou XL, Zhang MS, Zheng XR, Zhang ZQ, Liu JZ. 2024. Increasing the robustness of *Escherichia coli* for aromatic chemicals production through transcription factor engineering. Adv Biotechnol (Singap). 2, 15-25. doi: 10.1007/s44307-024-00023-x.
7. Koma D, Kishida T, Yoshida E, Ohashi H, Yamanaka H, Moriyoshi K, Nagamori E, Ohmoto T. 2020. Chromosome Engineering To Generate Plasmid-Free Phenylalanine- and Tyrosine-Overproducing Escherichia coli Strains That Can Be Applied in the Generation of Aromatic-Compound-Producing Bacteria. Appl Environ Microbiol. 86, e00525-20. doi: 10.1128/AEM.00525-20.
8. Fujita T, Nguyen HD, Ito T, Zhou S, Osada L, Tateyama S, Kaneko T, Takaya N. Microbial monomers custom-synthesized to build true bio-derived aromatic polymers. 2013. Appl Microbiol Biotechnol. 97, 8887-8894. doi: 10.1007/s00253-013-5078-4.
